# A network simplification approach to ease topological studies about the food-web architecture

**DOI:** 10.1038/s41598-022-17508-1

**Published:** 2022-08-17

**Authors:** Andrea Gini, Simona Re, Angelo Facchini

**Affiliations:** 1 School of Science, Scuola Normale Superiore, Pisa, Italy; 2 National Research Council of Italy, Institute of Geosciences and Earth Resources (CNR-IGG), Torino, Italy; 3 IMT School for Advanced Studies Lucca, Lucca, Italy

**Keywords:** Ecological networks, Ecological networks

## Abstract

Food webs studies are intrinsically complex and time-consuming. Network data about trophic interaction across different large locations and ecosystems are scarce in comparison with general ecological data, especially if we consider terrestrial habitats. Here we present a complex network strategy to ease the gathering of the information by simplifying the collection of data with a taxonomic key. We test how well the topology of three different food webs retain their structure at the resolution of the nodes across distinct levels of simplification, and we estimate how community detection could be impacted by this strategy. The first level of simplification retains most of the general topological indices; betweenness and trophic levels seem to be consistent and robust even at the higher levels of simplification. This result suggests that generalisation and standardisation, as a good practice in food webs science, could benefit the community, both increasing the amount of open data available and the comparison among them, thus providing support especially for scientists that are new in this field and for exploratory analysis.

## Introduction

Food webs, but most in general ecological networks, are often used to understand complex interactions in an ecosystem, providing insights about different ecological topics^1,2^. Paradoxically, a network simplification approach during preliminary analysis may ease unravelling and catching such complexity.

Trophic networks can be described as mathematical objects of graph theory applied to ecology, specifically to the trophic interaction. In a food web, each node represents a biological entity, most of the time a species. When two or more species take part in a trophic relationship, a directed edge link their respective nodes, starting from the prey and ending to the predator^3,4^. In this paper we refer to nodes as the biological entities regardless of their taxonomical rank, and we refer to edges as the directed trophic relationship sustained by two or more nodes.

As known, the entire system encodes in its topological architecture the complexity, and complex networks are precious tools to frame the essence of this phenomenon^5,6^. Although the analysis of these complex networks reveals fundamental insights that are otherwise missed, we need to take into consideration the accessibility and the existence of the datum itself^7–10^ to ease the use of this rich and complex information. A simplified network may be subject of over-simplification, thus losing meaning in its topology and in the application of many analysis techniques. We expected the loss of significance for many node level indices starting from the lumping of the Order rank, however, the loss of information is slightly shifted until the upper ranks, especially for the Trophic Level, i.e., the trophic index described further in this paper.

Today the interest in trophic networks continues to increase, reflecting an increasing number of datasets available in open databases. However, to date, the quantity of data seems not to be on par compared to other ecological subfields, mainly due to the difficulties of the data gathering process^11^. In our opinion, exploratory researchers in this field could be boosted with an aprioristic simplification. This phenomenon will probably mitigate in the future for all ecological networks, as eDNA metabarcoding techniques are becoming available to the majority of institutions, enabling faster and more accurate biodiversity status assessments^12–14^, and with an increasing focus on data obtained through citizen science projects^15–18^. However, as of now, networks built around literature data, observations and diet heavily rely on the input data of the nodes and edges.

Many ecological networks of different type^19^ (e.g. pollination networks, bipartite n., host-parasite n., interaction n., and trophic networks) can be easily found and downloaded from open databases. With regards to terrestrial ecosystems, trophic networks built around peri-urban and urban environments are scarcely represented. As can be verified by looking at the literature, where reviews, studies and meta analyses published as of today rarely cover multiple food webs in urban areas^11,20–23^. In addition, when considering the open databases like, e.g., WebOfLife^24^ and Brose et al. from 2019^25^, data focusing on urban areas are very scarce and the data reported are not enough for performing in-depth analysis. One factor contributing to the lack of ready-to-use trophic networks is the vast amount of time and effort demanded to build the food webs starting from direct observation or stomach contents^11^, with the need to ascribe the obtained entities to the most specific taxonomic ranks. Therefore, the widespread subdivision in multiple categories and the heteogeneous distribution of data^23^ contribute to a significant fragmentation of information from open repositories.

Many researchers identify food webs by trying to maintain the best possible taxonomic resolution for all taxa and limiting the upper ranks only to unidentifiable species. In our opinion, there is a pressing need to significantly speed up the data collection process, promoting the use of biodiversity data in novel urban areas. According to the literature, key topics that would greatly benefit from such data span ecosystem services evolution^26,27^, ecological security^28,29^, conservation^30,31^, land-use planning^32,33^, and ecosystems robustness and resilience^14,34,35^. In this sense, having food webs data from distinct urban habitats and geographic locations could be the basis for drawing comparisons between the trophic architectures of the ecosystems and integrating the knowledge provided by spatial models. Also, having many different food webs as open data with a unified and easily reproducible computation is critical^36^. It intrinsically explains how much networks are plastic and adaptable to our ecological studies.

Despite the massive spike of interest in the admixture between network science and ecology, many articles treat web building differently. It is undoubtedly better to have consistently high-resolution data both for nodes and edges^11^, but taxonomic aggregation is an already existing practice in network data on public repositories. This issue was extensively treated by Pringle and Hutchinson (2020)^11^ in their review, where they conclude the importance of resolution, especially for specialistic research. They also state that some ecological problems could be answered with a lower resolution based on past literature. In our humble opinion, having some sub-optimal data is better than having none, especially for exploratory or fundamental research. The availability of a simplified approach could benefit the comparison between food webs and facilitate scientists who want to approach this field for the first time.

Here we provide a framework to ease the data gathering process, at least for exploratory studies and provide good practices which can ease the comparison between different food webs. Using complex network^4,6,37^ node-level metrics that are considered common centralities indices, and an aprioristic simplification on the nodes taxonomy. We think that quick insights into the species connections and their relative importance for a habitat of interest could pave the way for future and more complex analysis. We have chosen different node level topological metrics that are ready to use in the most common network science libraries, both in R and Python: Degree Centrality (DC), Betweenness Centrality (BC), Closeness Centrality (CC), Trophic Level^38^ (TL), and Katz Centrality (KZ). These indices describe how connected a taxon is, whether it is important in connecting distinct parts or many different nodes of the food web, how close it is to the information flow of the graph and the hierarchy based on trophism. We are aware of the existence of indices and measures specific to ecology for macroscopic network analysis^39,40^, and extensive multivariable reports on indices^41–43^. Here, coherent with our aim, we have compromised on ready to use topological indices already computed in the past^44–48^ with a resolution on the nodes and not on the whole network, and comparable and complementary to each other topology-wise. Since food webs could be considered as strongly structured hierarchical networks^49^, we decided to measure also the general hierarchy of the graphs, together with the trophic levels^38^. We also included the clustering coefficient as a global measure to show general differences when the same network is manipulated multiple times^50^.

We calculated these measures on three food webs with distinct properties in order to test our approach with various inputs that are reasonably common to emerge from habitat surveys or for already accessible literature data, including a trophic network with a smaller number of nodes and a semi-disorganised taxonomy (North Carolina food web^51^), one with weighted edges (Caribbean food web^52^), and the third one with a larger number of nodes (Alaska food web^53^). Each food web is available through a trophic network open database (i.e., Web of Life^24^) in various formats or as supplementary material for previous research (i.e., Alaska food web^53^). The information about these datasets is retrievable in the Data availability section.

We calculated all the above measures to study how simplification affects the topology and how much structural information is thus encoded in the architecture. Then, we progressively simplified the three food webs by aggregating the nodes by taxonomy. Aggregation of nodes is not new in ecological network science; lumping nodes using taxonomy can be a choice or a necessity^11^, and it is known in the literature that simplified trophic networks (either by taxonomy or functions) retain some level of information about the original ones as global and local indices^54–57^, but this approach often uses aggregated values and a dedicated food web built for the sake of testing the simplification. Other types of aggregation have been used to compare the same food web at different time^58^. However, what we wanted to test in our study was whether all the single topological measurements that we choose at the level of resolution of the single node remain usable if nodes become aggregated, how community detection algorithms react to the simplified network, and if there were differences in performance for a different type of trophic network usually encountered. We also wanted to test how topological indices change puntiformly node by node after a taxonomical simplification and what is the trade-off of this technique. It is our opinion that a certain level of simplification coherent with the aim of the researcher not only could be taken a priori to speed up the framing of the project but also contributes to the plethora of ready-to-access, reproducible, comparable and sharable knowledge about ecosystems in which this type of data is not rich.

Because biodiversity is becoming more and more central for supranational laws and treates^59,60^, large-scale biodiversity assessments in urban and peri-urban areas are urgently needed in relation to their importance for the ecosystemic services and with the green transition. We think that complex networks could support the study of these webs of relationships across distinct fields, integrating the urban ecological knowledge^61,62^. Our simplification scheme can also be used as a metafile to easily share the level of taxonomical resolution for each taxa present in the network.

## Results

A general quantitative description (number of nodes, edges, average degree and density) of the three food webs at different levels of simplification is given in Table 1.

The collection of graphs, grouped by food web source and then by level of simplification, can be seen in the Supplementary document ‘Networks visualisations.pdf’. The readability of a network visualisation scales with the network dimension and is often problematic even for medium sized graphs. The “spring layout” scales the edges lengths and highlights the presence of more connected nodes, but is often prone to overlapped edges (Supplementary figure S7). On the other hand, in the “circular layout” all the nodes have the same positional relevance, but it helps to highlight the connections between the various components of the network (Supplementary figure S8), complementing the first visualisation. A red colour scheme highlights higher value nodes for the metric computed in this study.

We found that the topology tendencies of the least simplified network (i.e., namely “low” level) are generally maintained in all the three food webs, with a slight variation on a given measure of centrality that reflects the variety of the food web considered.

**Figure 1:**
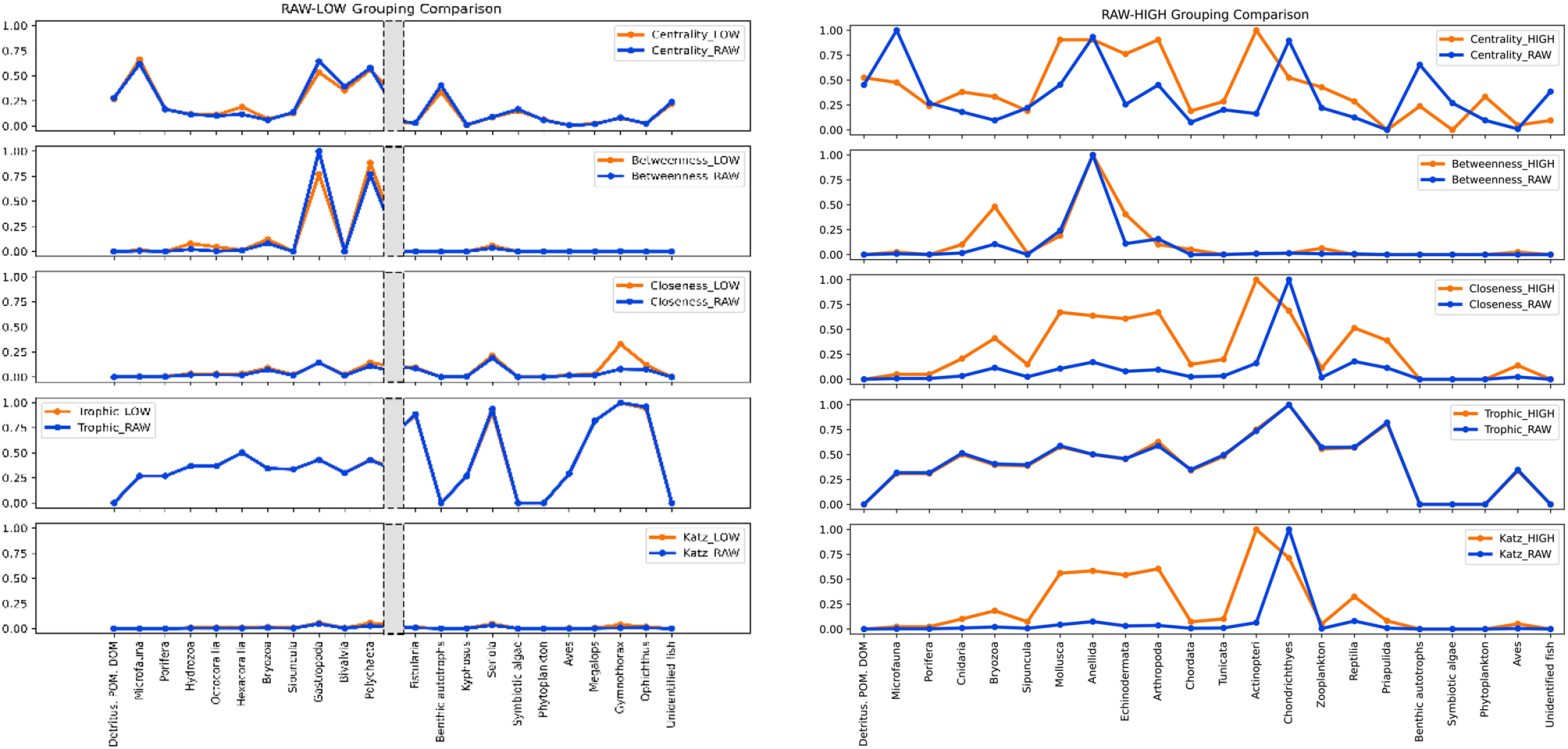
Caribbean food web's topology tendencies between raw data and low level of simplification (on the left) and high level of simplification (on the right). The left figure shows only the two extremities of the plot. For the complete visualisation (as well as the other levels of simplifications), see the Supplementary Materials. This is a categorical parallel coordinates plot, the point represents the actual data, and the lines help visualise how overimposable the values of the indices are. Blue dots and lines represent the index of choice (from top to bottom: Degree centrality, Betweenness centrality, Closeness centrality, Trophic level, Katz centrality) for the original network; orange dots and lines represent the same index for the simplified graph. In the Supplementary materials are reported all these types of visualisation for all the three food webs as vectorial .svg files.

North Carolina’s only simplified food web maintains an almost perfect trend overlap of betweenness and trophic levels and a good overlap of centrality values (see Figure S37). The Caribbean food web shows almost perfect overlap for all the different topology measures at the lowest level of simplification, with discrepancies in two grouped taxa (i.e., Malacostraca and Carcharhinus) but complete overlap in trophic trends. At the medium and high levels of simplification, we observed a progressive degradation of the information encoded in the network topology. However, the trophic levels remain perfectly overlapping, and the betweenness centrality is still comparable between the original and the simplified network (see Fig. 1).

The trophic levels of the Alaska food web, closeness and betweenness centrality remain comparable to the original from low to medium level, with the former remaining consistent even at a higher level of simplification. Nodes centrality is not a good indicator for this particular network, and from the high level of simplification, all the measures, except trophic centrality, begin to lose meaning and cannot be used as good indicators of topology (see Figures S27-31).

**Figure 2:**
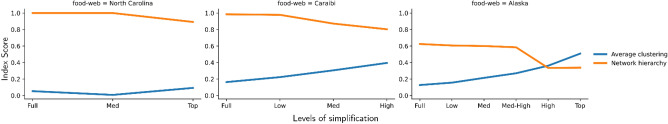
Average clustering and hierarchy trends amongst food webs and levels of simplification. Each of the three plots shows with a common index score (0–1) how much the average clustering and the network hierarchy change for a given food web, increasing the simplification and lowering the taxonomic resolution. For example, in the Alaska food web, the “row”, “low”, and “med” levels maintain these two measures till the “med-high” level, in which further simplification degenerate the network. At the “top” level, the majority of the nodes become connected, and the two indices overlap and switch positions.

In all networks, we observed an opposite trend in clustering and hierarchisation while the level of simplification increased (see Fig. 2 and Table S1).

**Figure 3:**
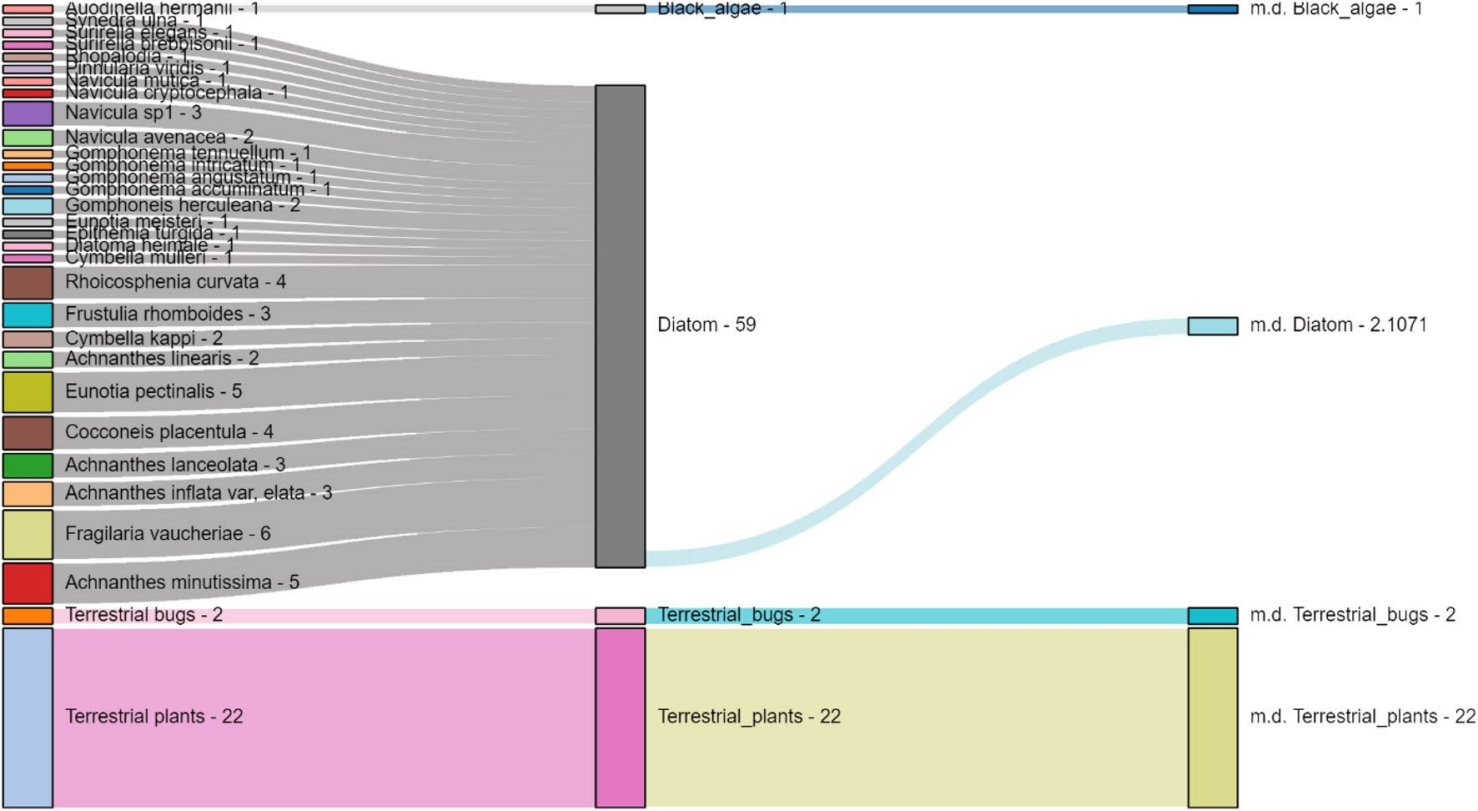
Crop of the Sankey visualisation for the flow of information about the node degree between the raw graph and the grouped network. In the first column, all the original nodes are listed with their own degree; in the middle one, the sum of their degrees identifies the belonging group in the grouped network; in the last one, we show the mean degree (m.d.) of the group based on the data of the original network. The complete visualisation, as well as the other Sankey interactive plots, can be seen in the North_Carolina_Sankey.7z archive in the Supplementary material as HTML files.

The modified Sankey diagram reported in Fig. 3 (the interactive view is attached as Supplementary figure S38 as for all other views) shows the flow of node degree raw and mean values.

The most visible evidence concerns the clustering effect on diatoms. They collectively achieve a cumulative amount of 59 in out-degree and an average degree of 2.1; in the simplified network, they collectively achieve a degree of 11. In contrast, terrestrial plants have the mean degree equal to their node degree (because they are represented as a single node in the original graph) and, in the clustered graph, reach a value of 14. In Table S2, we have reported the degree values (i.e., the number of incoming and outcoming edges per node) for the original and simplified network.

**Figure 4:**
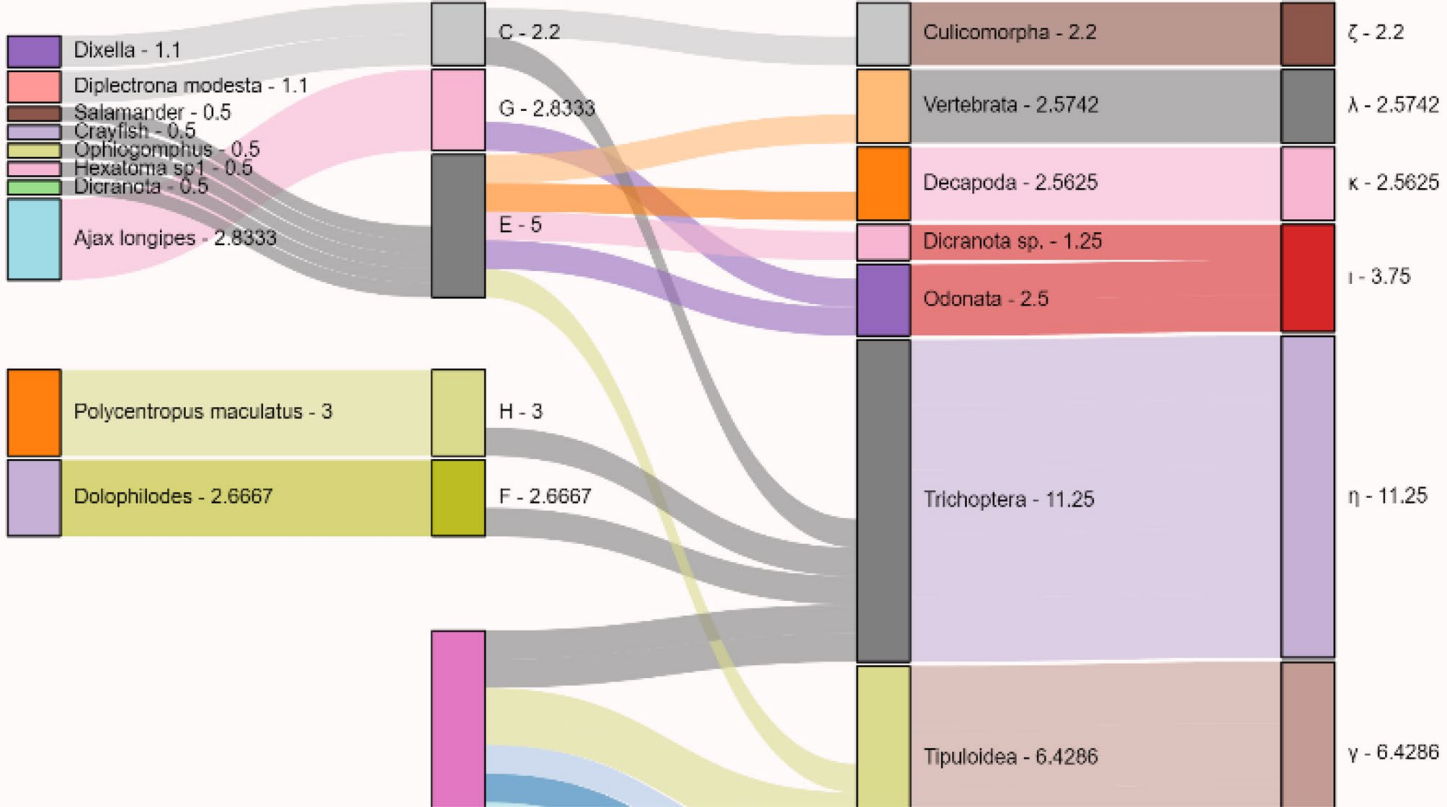
Extract of the North Carolina Sankey visualisation for trophic levels flux. The columns represent, from left to right: the Trophic Level (TL) in the original network (ON) subdivided by the number of entities belonging in a cluster; the clusters based on the same value of TL in the ON; TL computed on the simplified network (SN) subdivided by the number of entities belonging in a cluster; clusters based on the same value of TN in the SN. The values near the organisms indicate the TL of the node. The height and the number of the second and third columns are the sums of the TL in the same cluster (with a minimum value set to 1 to optimise the visualisation). For the complete interactive Html output, see the Supplementary figure S42.

The trophic clusterisation depicted in Fig. 4 shows the consistency between the original graph (i.e., Latin letters) and the simplified network (i.e., Greek letters). While the segregation for primary productors is the same (letter A to α), predators in cluster E with the same value (trophic level 2.5) all segregate into different clusters in the simplified network. In this case, the network with the clustered nodes remains more granular and differentiates each of the predators, with different trophic values, into distinct clusters.

For the other type of clusterisation and community detection, we obtain similar results between the original network and the grouped one.

Katz-centrality performs very similarly to the trophic clustering but with a more pronounced granularity on the number of different groups in each network.

The directed Infomap algorithm performs better in the simplified network, as it recognises a more accurate separation with biological significance between different taxa. The middle layers of the Sankey diagram are clearly intertangled. The Sankey visualisation for both the Katz centrality and the directed Infomap algorithm can be seen in the Supplementary material (Figure S40-41).

The Girvan-Newman algorithm produces three clusters in both networks, but the original one divided the diatoms taxa into all three different groups; the Sankey visualisation and dendrograms are shown in the Supplementary materials (respectively Figure S39, S35 and S36).

Analysis of the goodness of clustering for trophic levels, Katz-centrality and the Infomap-directed algorithm are shown in Table 2. Infomap performed very similarly in the original and simplified graph and is comparable in effectiveness in recognising the same group of taxa with robust biological significance.

However, future studies are needed to assess how all the other features of complexity perform differently in a grouped network. We found a granular detection of clusters linked by taxonomy, but the original groups are harder to be found in the manipulated food web.

## Discussion

The three food webs of our choice could be generalised as three common patterns of data that might be expected or found when building a trophic network: inconsistency of taxonomy resolution across all nodes and a lower number of species (North Carolina); a large and a challenging to manage a number of species (Alaska); a trophic network that is weighted (Caribbean). In all these three cases, we show that the hierarchy, betweenness and trophic levels are maintained if the species are at least grouped at the genus level. However, these three measures are also robust and consistent where simplification reaches the family and the order levels. The “low” level of simplification maintains almost perfect topological information with all the general indices of our choice, with differences between food webs due to the nature of the data itself.

We notice—as a by-product—that simplifying the original food web could significantly help in communicating the relative importance of a species and its group. For example, the loss of a single diatom species in the North Carolina network poorly reflects the cumulative degree. In contrast, the loss of a species mainly or entirely contributing to the degree of its group has a greater impact on the topology of the food web. Consequently, its loss makes it reasonable that the trophic architecture becomes prone to cascade effects. In the Sankey flux visualisation in Fig. 3, it is visible that the most represented group, diatoms, represents only 2.1 degrees, as opposed to Ectopria thoracica, which reaches a degree of 15. Along the same line of reasoning, this way of visualising and implying data could be a valuable strategy to underline how vital biodiversity is in maintaining the resilience of an ecosystem and absorbing stresses, globally sustaining its robustness.

In general, the recalculated degree value for the simplified network mediates the trend of the degree and the average degree from the past example (e.g., a single diatom contributes poorly to the network’s stability, but taken together, they are the foundation of the food web).

The Caribbean network has a high number of species that could be grouped by genus while maintaining the hierarchy. It does not contain many microscopic entities, and the number of connections suggests that each node is connected several times. Thus, the low level of simplification seems to be a useful substitute for inferring general topological features, decreasing the time required to build a large graph at the resolution level of the species.

Only the trophic levels and the betweenness centralities of the Alaska food web are robust. They could be used to perform a quick analysis of the network’s structure without investing much time in resolving the taxonomy of the species. However, our approach does not work effectively with networks that contain many loops and a high number of microscopic entities, such as this one, because the division between predators and productors is very thin. We think that food webs researchers who want to focus and preserve information on microbial loops and taxa of microscopic entities need to maintain as high a taxonomic resolution as possible, or at least compartmentalise their research into two primary investigations regarding the microscopic and macroscopic scale of their habitat of interest.

The North Carolina food web could be considered as an example of a network where data are not systematically recorded or a food web not developed directly on the field but with previous data. Some entities are resolved to the species level, but globally taxa range from genus to common name. Another potential issue is that many different species are in distinct groups. However, each group often contains only a few entities so a specific trophic level may be characterised only by that minority. The use of a mixture of commonly known higher taxonomic ranks (e.g., superfamily Tipuloidea) and vernacular names could help generalise the species groups further and communicate their importance to the public. In our opinion, it is imperative, especially in this case, to standardise the taxonomical resolution with a scheme to facilitate future comparison and meta-studies.

Our conclusion is that with these general topological indices, we basically cannot find any differences between original data and simplified data at the lowest possible level of simplification. From the data perspective, computing indices from the “raw” or the “low” level of simplification are the same. An aprioristic approach could speed up the process if a researcher wants to make an exploratory analysis or compute only these indices as in previous research^44–48,63^.

The Sankey graphs for the clustering algorithms in the Supplementary material are more organised, and less intertangled for the simplified network. Overall similar trophic groups are not segregated into different clusters. Simplified trophic networks could be used to compare the existence of clusters and communities between the raw granular data and the simplified entities. Despite our results with the topological indices where the simplification process could be used to facilitate the analysis, we must stress that for the evaluation of communities, it is useful to compare the original with the clustered data in order to assess how the clustering process differs and behaves between the original and the simplified network. Community detection and clusterisation is something that, in our opinion, should be performed in order to simplify or aggregate data and, therefore, not to compute on already simplified data. Despite the greater organisation of the grouped network, the number of clusters differs with different algorithms. Thus, simplification does not increase the consistency between the expected and obtained clusters.

Although the interest in food webs and the increase in available open network data, there is still a scarcity and unevenness of datasets. Many easy-to-reproduce ecological graphs fall into the subcategory of food webs, pollination networks and host-parasite relationships. Of the many food webs easily reachable through open databases or libraries, most are focused on either marine and freshwater habitats. Exploring Mangal^64^, WebOfLife^24^ and the Supplementary Table 1 in Brose et al. from 2019^25^, terrestrial environments with a high number of taxa listed, especially anthropic impacted locations, remain in the minority. Zones around and inside peri-urban and urban areas are critical in regard to the thinning of their ecological network, and they need multiple studies to further the knowledge. However, it is our thinking that these locations represent key zones to study the effects of urbanisation on the ecosystem, and exploratory analysis with simplified webs can serve the purpose of a trigger to more profound future research. It is only recently that the industrial field tried to look at ecological networks to compare the artificial performance of a manufactured web against a natural network^65^. In conjunction with landscape ecology, we think these two fields need to be concerted for studying the urban environment as a whole^61,62^.

We concur with Pringle and Hutchinson (2020)^11^ regarding the quality and the insights on high-resolution food webs. As we stated in the introduction eDNA metabarcoding will play a key role in this matter, but as of today, we believe in a preventive approach to fill the network knowledge for anthropic impacted locations. Reasonably simplified complex networks remain complex systems and retain in their architecture important topological information with generalist centrality indices. We think that in this case, having simplified data is better than having none, especially as a starting point and as a trigger to future research. The lack of data is mainly attributable to the efforts, such as the number of people and hours required to construct a trophic network based on direct observations or stomach contents^11^.

Based on our findings, we propose to generalise the concept of a “network of species eating other species” to a “network of taxa” for early exploratory analysis. Despite the concept of “trophic group” or “trophic species”, most data are commonly classified at the highest possible level of taxonomic resolution, which also leads to a disparity in treating all the taxa with the exact resolution when drawing a comparison between networks. Suppose a simplification process is adapted in advance to the experiment and not a posteriori to deal with unknown entities. In that case, the resulting food webs may be more robust compared with other ecological networks in similar habitats.

We humbly suggest setting up a scheme as a good practice when simplification is based on taxonomy, at least for preliminary research or trophic studies that span large areas with many diverse organisms. In this way, if a researcher limits its analysis to basic topological indices, the information about the food web architecture remains consistent and comparable, taking away the experimental design liability from the researcher. A meta-file scheme used as a descriptor or as a supplementary file could help in matching other networks at the same taxonomical level. Particular attention must be placed on the scope of the analysis. If plants or insects are what the researcher wants to investigate, they can be treated as the vertebrates in our “low” level of simplification. We do not mean to limit the vast amount of methodologies possible to study a high-resolution network, but we aim to stress how much is essential to increase the number of generalised data about species interactions in anthropic impacted habitats. Nevertheless, we believe that adhering to a standard scheme could benefit this field in two ways: vertically, by easing the initial stages of the network building; horizontally, by facilitating the comparison between different networks with a supplementary meta-file.

We found that the taxonomic information present in some databases is different or less updated; this could hinder the comparison, especially with plants and arthropods. In our opinion, using a simplified approach at the genus or family level strongly reduces the data cleaning process done by researchers when dealing with the taxonomic data from different sources. This could be even more important in the case a networked representation is used by a technician or policy maker not updated on the taxonomy like experts and scientists. When a food web is built around past data or previous research in which the source material is not a network already, aprioristic simplification could help authors of meta-analysis in generalising their node entities across a multitude of past studies.

In conclusion, we assessed at the node scale that network simplification by taxonomical grouping could be used as an exploratory method with general topological indices. Speeding up the network building phase and potentially attracting novel researchers in producing more network data. This method needs to be contextualised with the scope of the research because microbial loops and community detection need a higher taxonomical resolution. Over-simplification is a problem. As is clearly visible in Figs. 1, 2 and in Supplementary figures S27-34, when nodes are lumped together to the extremes of the taxonomical ranking (Class, Order and Phylum) the loss of complexity and information is excessive. An extreme simplification may be the origin of absurdities. If very different biological entities get grouped in the same node, it is theoretically possible to have a predator and its own prey in the same node. We believe that a simplification approach can be extremely useful, but only if it is performed with reasoning a common sense as criteria. In this study we voluntarily took the nodes grouping to an extreme because we wanted to find where the simplification starts to break the topology. We placed a reasonable trade-off between simplification and robustness from the “low” to the “med” level of simplification. Thus, we suggest as a rule of thumb, to limit the node lumping to these levels (focusing on the group of species of interest as shown in Table 3), and to simplify as much as the Genus rank for the taxa of interest.

**Table 1:** Network summary for the three food webs nested per level of simplification. Raw indicates the network as downloaded and built. Low, med, med-hi, high, top and grouped represent different levels of simplification (see Methods). Besides the number of nodes and edges of the three food webs per level of simplification, we provide the average degree and the density. The average degree is general measure of connectedness and shows the average number of edges per nodes. Density expresses the ratio of the existing connections and the number of maximum edges that could be present inside a generic graph (0 means that a graph does not contain any edge, and 1 represents a complete graph). Each of these four measurements were computed for every food webs at every level of simplification.

	North Carolina		Caribbean				Alaska					
	Raw	Grouped	Raw	Low	Med	High	Raw	Low	Med	Med-hi	High	Top
Number of nodes	71	25	250	154	64	22	513	392	289	196	112	30
Number of edges	148	72	3355	1734	594	117	6774	5237	3684	2367	1191	216
Average degree	2,08	2,88	13,42	11,26	9,28	5,32	13,20	13,36	12,75	12,08	10,63	7,20
Density	0,03	0,12	0,05	0,07	0,15	0,25	0,03	0,03	0,04	0,06	0,10	0,25

**Table 2:** North Carolina clusterisation performance between original and simplified food web. We reported each measure of clusterisation performance for the original (left) and the simplified (right) NC food web. The three sub-columns indicate how the community detection algorithm or the index used for the cluster creation performs.

	NC Graph			NC Grouped_Graph		
	Infomap	Katz	Trophic	Infomap	Katz	Trophic
Rand index	0,54	0,85	0,86	0,54	0,74	0,75
Homogeneity	0,24	0,81	0,73	0,19	0,84	0,73
Completeness	0,10	0,40	0,57	0,17	0,40	0,40
V-measure	0,14	0,53	0,64	0,18	0,55	0,52
Fowlkes Mallows score	0,24	0,79	0,82	0,32	0,47	0,52

**Table 3:** Taxonomic ranks across levels of simplification for the Alaska and Caribbean food webs. In our case, we decided to focus on fishes (or vertebrates in general) for both of these networks, so this group is simplified at the genus level, both at the “low” and the “med” levels. This choice must be contextualised to the major taxon or taxa of interest (e.g., pollinator insects in a pollination network).

	Alaska					Caribbean		
	Low	Med	Med-high	High	Top	Low	Med	High
Chromista	Order	Class	Class	Class	Kingdom			
Echinodermata	Family	Order	Order	Class	Phylum	Class	Class	Phylum
Chordata	Genus	Genus	Family	Order	Class	Genus	Order	Class
Mollusca	Family	Order	Order	Class	Phylum	Class	Class	Phylum
Plantae	Order	Class	Class	Class	Phylum			
Protozoa	Order	Class	Class	Class	Phylum			
Anellida	Family	Order	Order	Class	Phylum	Class	Class	Phylum
Arthropoda	Family	Order	Order	Class	Phylum	Class	Class	Phylum
Nematoda	Family	Order	Order	Class	Phylum			
Cnidaria	Family	Order	Order	Class	Phylum	Class	Class	Phylum
Platyhelminthes	Family	Order	Order	Class	Phylum			
Porifera	Family	Order	Order	Class	Phylum	Phylum	Phylum	Phylum

We are convinced that a common collaborative way of generalising a food web could be beneficial as a preparatory and rapid analysis and promote the exchange of information and the comparison of different food webs adhering to a meta-file as a best practice. We are not suggesting reducing the quality of a large and precise network, aggregating something that potentially could hide some topological importance. Simplification by taxonomy is an already used approach and, with some standardisation, this could become not a hindrance but a useful tool to speed up the data collection process, ease the sharing of ecological networks by means of accessibility and, at the same time, to entice new researchers to the food-webs topic.

## Methods

### Taxonomic simplification of original datasets

The North Carolina (FW_012_02) and Caribbean (FW_008) food webs are retrievable in several formats from the Web of Life^24^ database using the FW codes. The Alaska dataset can be accessed in the Supplementary Data of the original article cited in the introduction.

The North Carolina food web was already grouped at a different taxonomic level, so we proceeded manually, simplifying it with a mixed approach. We re-grouped entities taxonomically (e.g., Salamander as Vertebrata) and by “common words” to highlight potential differences between the terrestrial and aquatic compartment (e.g., *Ectopria thoracica* as Beetles_water). As the different entities at the species level of a genus were mostly present once per genus, and as the food web was already small in taxa, we decided to use the common names of the higher taxa group to simplify the output and to maximise the readability of the grouped network. Undetermined species are named und in the results.

The Caribbean and Alaska food webs were rich in species of the same genus and abundant entities, so we automated this process. We used the Python porting (still under development by the original authors) of the R package taxize (v. 0.9.99)^66^ to retrieve the taxonomic hierarchy. The Caribbean taxa list was checked against the NCBI taxonomy database, the Alaska taxa list provided TSN codes, and then we checked the hierarchy against the ITIS database. We grouped taxa with different resolutions creating the simplified food webs LOW, MED, MED_HIGH, HIGH, and TOP, where each level of simplification is a simplified version of the predecessor. For entities with common names, we kept the taxon name as high as possible (see Table 3 for a list of the simplifications of the main groups in common for both networks). All the taxonomic datasheets are present in the Supplementary material, as well as the raw lists and with the node lumping in a spreadsheet format to broaden the audience.

We choose this simplification scheme to reflect the interest of the researcher could be. For example, in a fisheries study, it is reasonable to maintain a high resolution of fishes. The type of simplification depends totally on the aim of the researcher, but we suggest simplifying as much as the genus for the lowest level of simplification for the main taxa (e.g., insects in a pollination network or mammals in a Theria behavioural network). What is really important is to adhere to a scheme in order to better share the level of resolution used in the building process.

### Networks analysis and topology

Each food web was rebuilt via NetworkX (v. 2.5)^67^ as directed weighted networks. The raw data from the North Carolina and the Alaska food webs were unweighted, so weights have been considered only for the simplified grouped graphs. We rebuilt all the edgelists at different levels of simplification by mapping the original data to the new simplified taxonomy, resulting in lists of the same length with repetitive values. We assigned as weight the sum of the original weight (considered 1, if unweighted) for entities that became identical after the simplification process. De facto merging taxa to have only one predator–prey interaction per network (see Table 4 for a theoretical example).

**Table 4:** Example of the simplification process. The first three columns refer to an original network, and the last three refer to a simplified version of the original one. The weight for Podarcis eating insect of Genus B adds to 2 because when grouped, a duplicate of Podarcis eats the same insect B when the species is stripped of the specific epithet.

Predator (raw)	Prey (Genus)	Weight (raw)	Predator (simpl.)	Prey (Genus)	Weight (simpl.)
*Podarcis muralis*	Insect A	Weight = 1	*Podarcis*	Insect A	Weight = 1
*Podarcis muralis*	Insect B	Weight = 1	*Podarcis*	Insect B	Weight = 2
*Podarcis siculus*	Insect B	Weight = 1			

We used nodes' degree centrality, betweenness and closeness as reliable indicators of each node’s position and influence on the other nodes because they are straightforward to compute in most libraries. They were also extensively treated with their bad and good use case scenario in literature^50,68^. Being similar and of the same ‘family’^41,69^, these indices can also be compared and analysed with the same simplification procedure.

The node degree, and so the Degree Centrality (DC), express in how many connections a node takes part and it is the normalised sum of the incoming and outcoming edges. The Betweenness Centrality (BC) measures the number of shortest paths (i.e., a path between two nodes that minimise the passage costs over edges) passing for a node. Closeness Centrality (CC) express how moch close a node is to all other nodes, namely the reciprocal of the sum of the lengths of the shortest paths passing for that node and all the other nodes of the graph. The Trophic Level (TL), or “trophic position”, ranks the nodes based on an energy budget model and on the flow of this energy from producers to consumers^38^.

According to the concept of the node position and relative importance, we chose Katz Centrality (KC) because it measures the importance of a node based on its directly connected neighbours and their connection with all the other nodes in the network. It is an application of the eigenvector centrality that solves the issue of its computation on directed networks by giving a certain amount of centrality for free^37^. Spectral eigenvector centralities (EC) were previously investigated by Allesina and Pascual in 2009^70^.

Average clustering and network hierarchy represent respectively how much the nodes of a network are connected and how much the flow of the connections are in a hierarchical structure avoiding cycles.

These node level indices and general measurements, though more used in the Social domain of Network science, were investigated also in recent research of the ecological field (see Supplementary Table S3 for detailed list of metrics and previous researches).

Results were min–max [0,1]^71^ normalised (Eq. 1) to compare values between different groups with Pandas (v. 1.2.3)^72^, and each visualisation was calculated with Matplotlib (v. 3.3.4)^73^ and Seaborn (v. 0.11.1)^74^.

1 $$x^{\prime} = \frac{{x - {\text{min}}\left( x \right)}}{{\max \left( x \right) - {\text{min}}\left( x \right)}}$$

Node degree, centrality, betweenness, closeness, trophic level and Katz centrality were computed with NetworkX. We did not analyse Katz's centrality for the Alaska food web due to the high number of nodes and inherent redundancies in this trophic network. We chose Girvan-Newman^75^ and Map Eq. ^76^ for community detection, and we decided to cluster also by trophic levels and Katz centrality. Trophic levels and their derivatives are commonly used as the starting point for detecting clusters within food webs^77,78^. Similarly, Katz centrality, in our opinion, could be a useful approach to discern in a more granular way group of species that have similar contracts with other taxa. An in-depth summary of the clustering approach is given in the final section of the Methods.

### Visualising the information flow

Among the three raw networks, we chose to further analyse the North Carolina food web. This particular network is easy to manage graphically due to the small number of entities present, is the most diverse in terms of species and habitats, and therefore most suitable to be studied for clustering performance. We adapted the use of the Sankey family visualisations (Sankey sensu stricto and alluvial plots) to visually follow how different metrics respond to our grouping technique. The two types of plots we arranged are for simple metrics (node degree, trophic level, etc.) or for community detection and clustering. In both cases, we listed the origin and destination of all nodes, as well as the value of interest. For the clustering flow, we divided the original values (e.g., the trophic level) of each taxon per number of taxa in a specific cluster in order to preserve a visual consistency between clusters’ sizes (e.g., 5 entities with a trophic level of 2.5 each were evaluated with a level of 0.5 to maintain their membership to the 2.5 cluster when lumped together). *With regard to the Sankey clustering visualisation*: the first column represents the original network; the second shows the cluster to which a group of nodes belong, with the size of the input lines showing the relative value of interest for that specific cluster (only for the second column, the block size is not always coincident with the input lines for clarity of visualisation); the third column represents the simplified taxa, and the fourth the clusters and their values for the clustered taxa. We assigned the standard value 1 to all middle fluxes (second and the fourth column) to better show all the links without a drastic change in the scale of the figure. This is visible in Fig. 4 where the predators reaching trophic level 2.5 and an assigned value of 0.5 each converge in a cluster of value 5 for five outbound links. Data for visualisation were handled with Pandas, and plots were computed with Holoviews (v. 1.14.2)^79^.

### Cluster analysis

We manually created diversity groups with the same biological significance to test the goodness of the clustering methods on the North Carolina food web. We did not take into account the function the organism has within the ecosystem because this could be different for the same species between different locations. So we chose to remain general, and each of our expected groups reflects a possibility of being a predator or a prey only for species present in the food web (1: productors; 2: predators that could be a prey; 3: top-predators; the full list is available in the supplementary material).

We calculated the Rand index, homogeneity, completeness, V-Measure and Fowlkes-Mallows score for three different sets of groups based on the Infomap algorithm, Katz centrality and trophic levels. These scores measure, respectively, how clustering performs between technique or with predefined standard labelling (i.e., true/expected clusters); whether data from a cluster are present in a single expected group and vice versa; a combination of homogeneity and completeness; and the similarity of the two groups of clusters considering true positive, false positive and false negative. All of these measures range from 0 to 1, where 0 means non-coherence and 1 stands for a perfectly matched labelling. The analysis was conducted with the Python library Scikit-learn (v. 0.24.1)^80^.

All analyses in this research were conducted in Python (v. 3.9.2).

## Supplementary Information

Supplementary Information 1.

Supplementary Information 2.

Supplementary Information 3.

Supplementary Information 4.

## Data Availability

Each food web is already open source and published on the network database provided in the introduction. In particular: the North Carolina food web is originally reported by Thompson, R. M. and Townsend, C. R.^51^ (http://www.web-of-life.es/map.php?type=7 searching for network FW_012_02); the Caribbean food web is originally reported from Bascompte J., Melián C. J. and Sala E.^52^ (http://www.web-of-life.es/map.php?type=7 searching for network FW_008); the Alaska food web is originally reported from Dunne J. A. et al.^53^ (in their Supplementary Data S1). Any data contained in the food webs are open for use but not owned by us.
