# Supplementary material for: A network simplification approach to ease topological studies about the food-web architecture
Source: Sci Rep. 2022 Aug 17;12:13948. doi: 10.1038/s41598-022-17508-1 (PMC9385703; doi:10.1038/s41598-022-17508-1)
Supplement: Supplementary file 1 — Supplementary Information 1. [file 41598_2022_17508_MOESM1_ESM.pdf]

Supplementary material I:

Food web networks per level of simplification

Color scheme: YlOrRd

Color changes from light yellow to dark red when the index value increments

List of Figures

S1 North Carolina RAW food web: Spring layout 2
S2 North Carolina Grouped food web: Spring layout 3
S3 North Carolina RAW food web: Circular layout 4
S4 North Carolina Grouped food web: Circular layout 5
S5 North Carolina RAW food web: Tree hierarchy 6
S6 North Carolina Grouped food web: Tree hierarchy 7
S7 Caribbean RAW food web: Spring layout 8
S8 Caribbean LOW food web: Spring layout 9
S9 Caribbean MED food web: Spring layout 10
S10 Caribbean HIGH food web: Spring layout 11
S11 Caribbean RAW food web: Circular layout 12
S12 Caribbean LOW food web: Circular layout 13
S13 Caribbean MED food web: Circular layout 14
S14 Caribbean HIGH food web: Circular layout 15
S15 Alaska RAW food web: Spring layout 16
S16 Alaska LOW food web: Spring layout 17
S17 Alaska MED food web: Spring layout 18
S18 Alaska MEDHIGH food web: Spring layout 19
S19 Alaska HIGH food web: Spring layout 20
S20 Alaska TOP food web: Spring layout 21
S21 Alaska RAW food web: Circular layout 22
S22 Alaska LOW food web: Circular layout 23
S23 Alaska MED food web: Circular layout 24
S24 Alaska MEDHIGH food web: Circular layout 25
S25 Alaska HIGH food web: Circular layout 26
S26 Alaska TOP food web: Circular layout 27

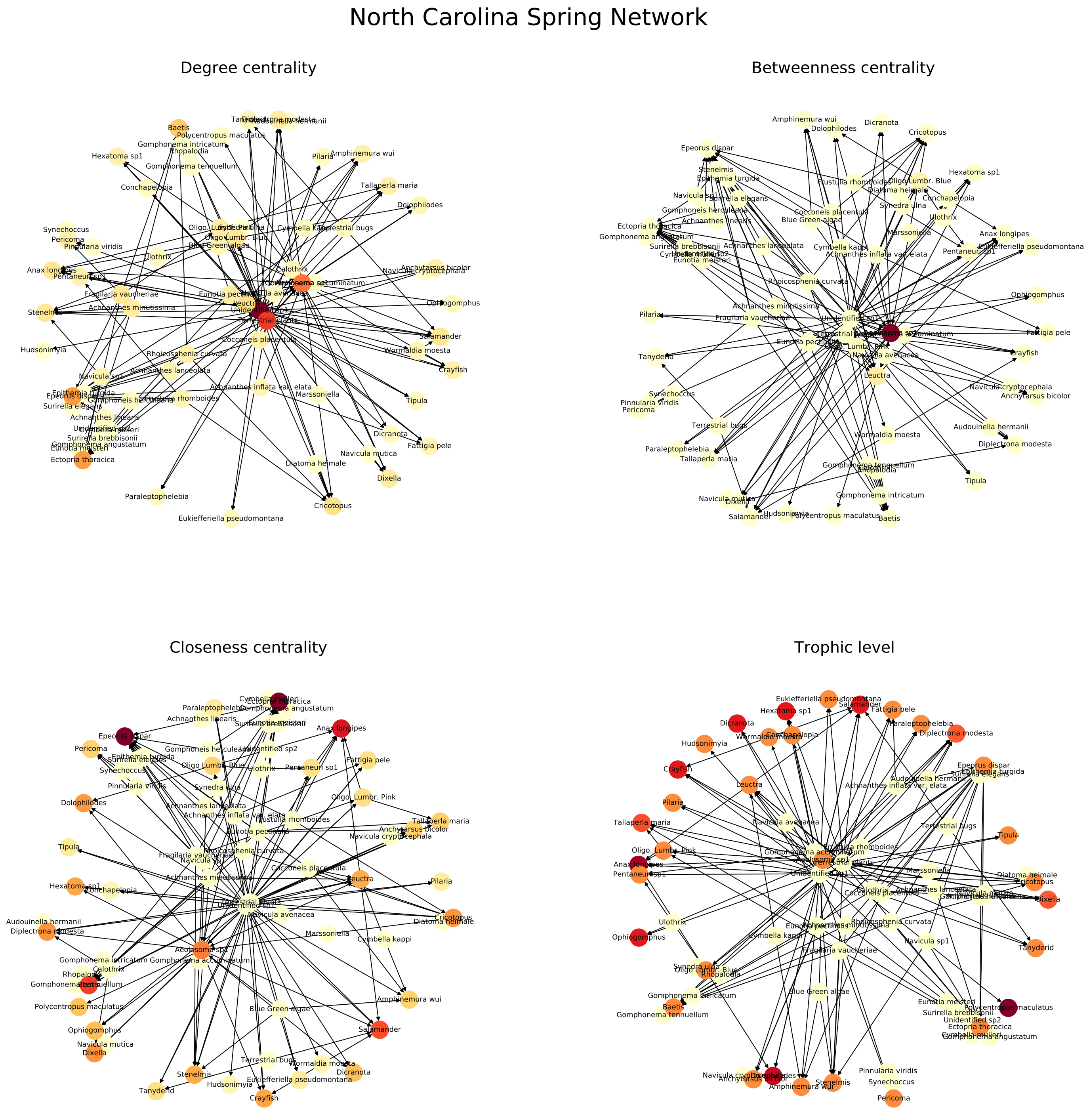

Figure S1: North Carolina RAW food web: Spring layout

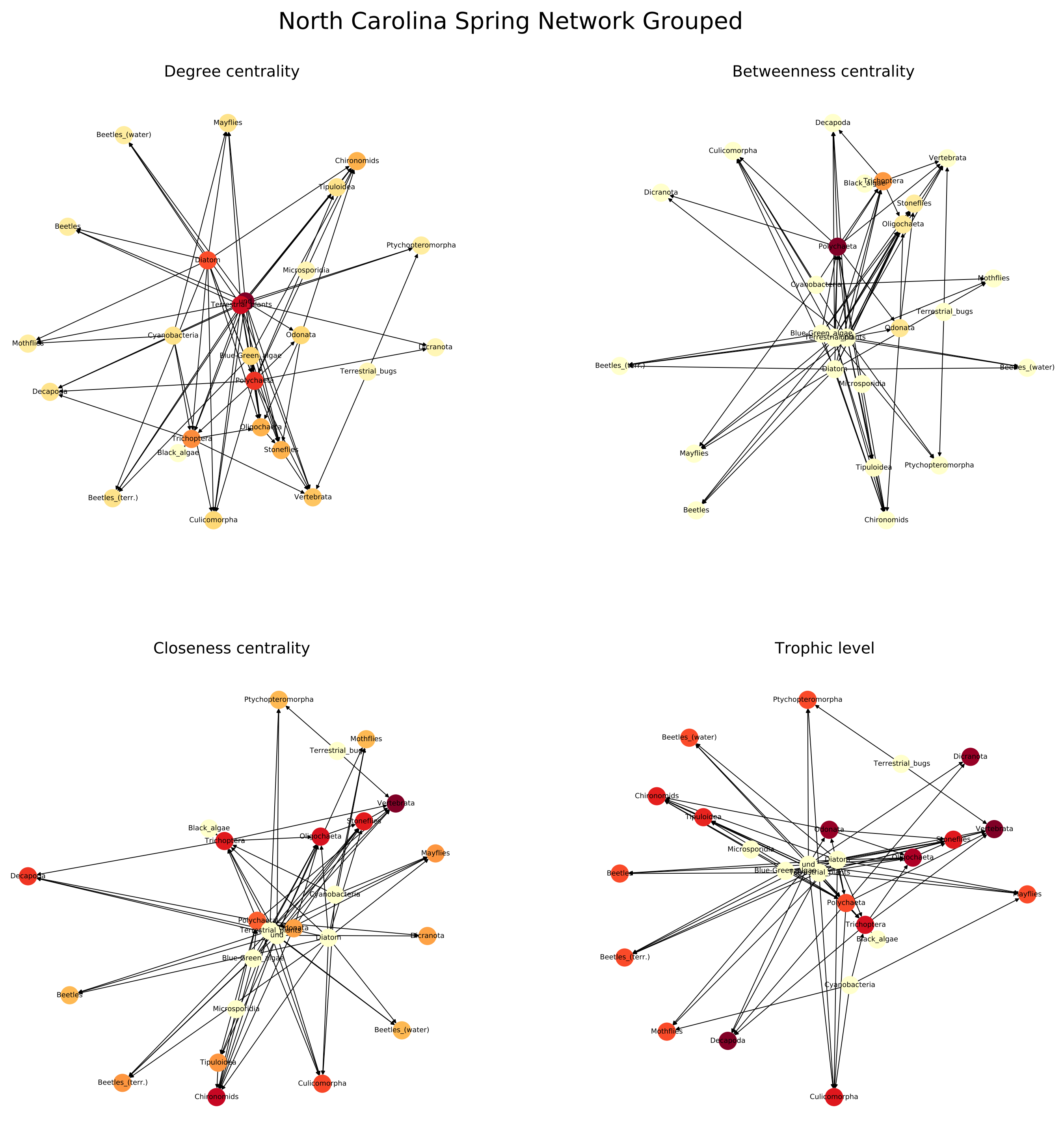

Figure S2: North Carolina Grouped food web: Spring layout

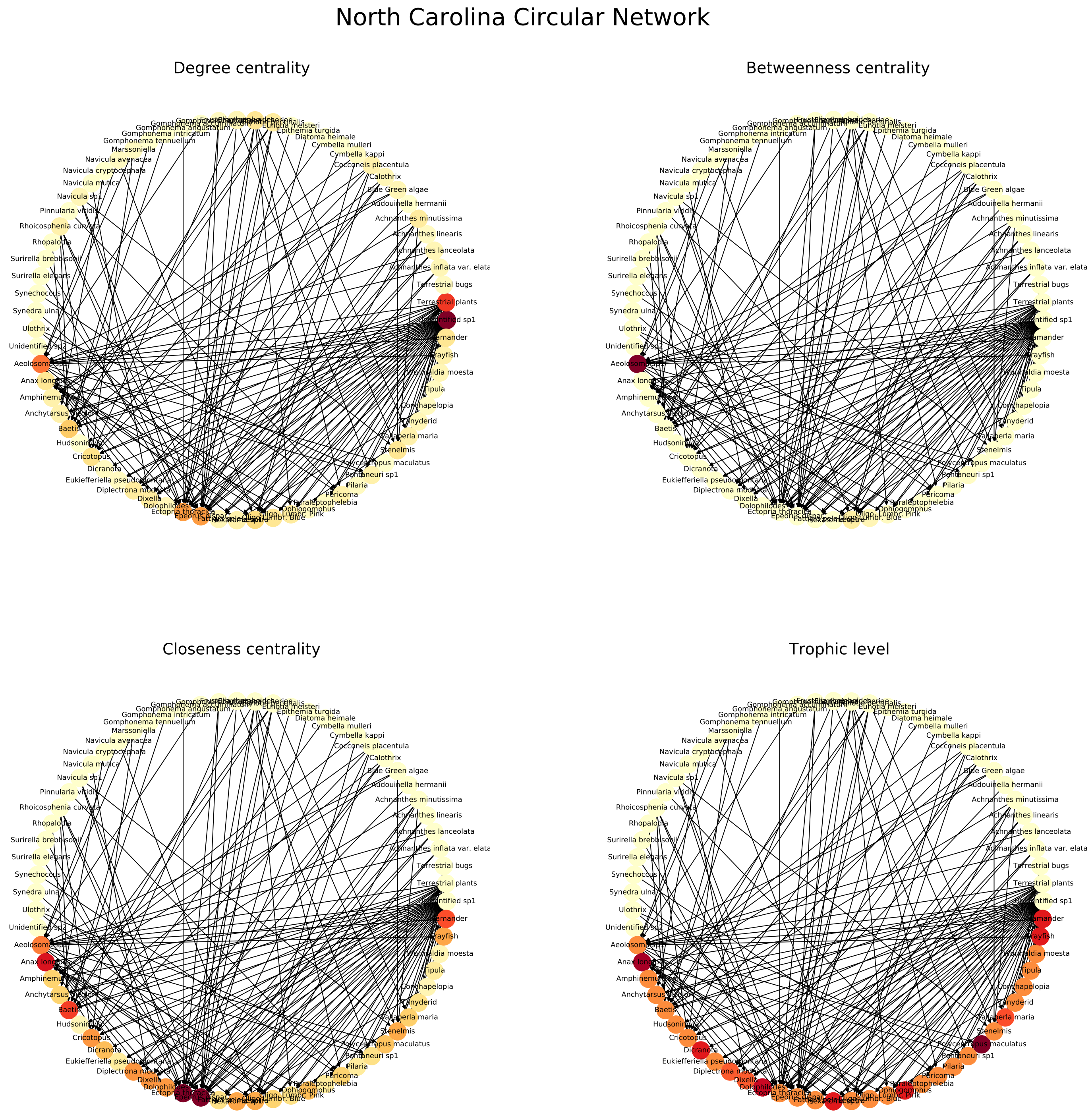

Figure S3: North Carolina RAW food web: Circular layout

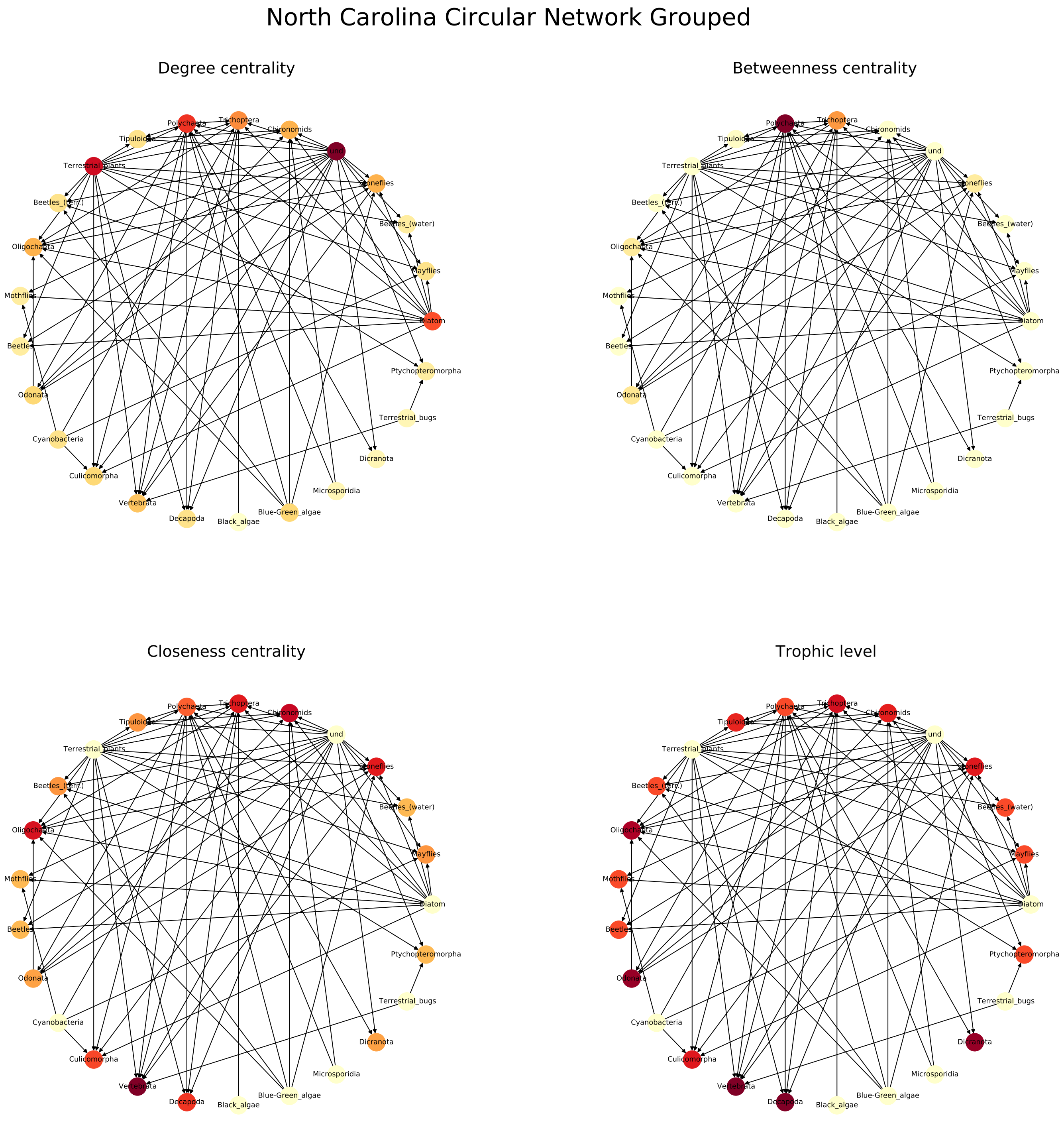

Figure S4: North Carolina Grouped food web: Circular layout

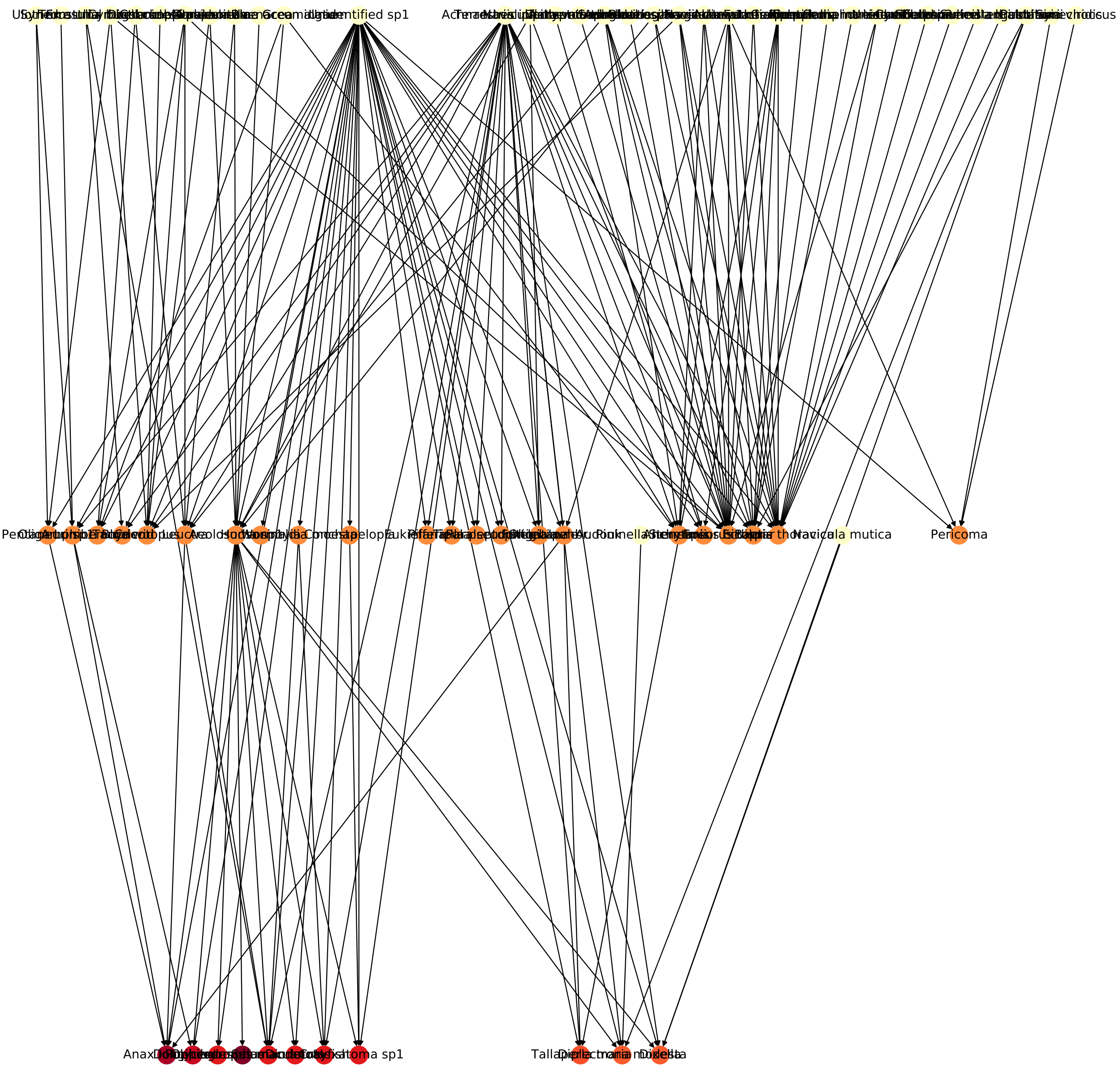

Figure S5: North Carolina RAW food web: Tree hierarchy

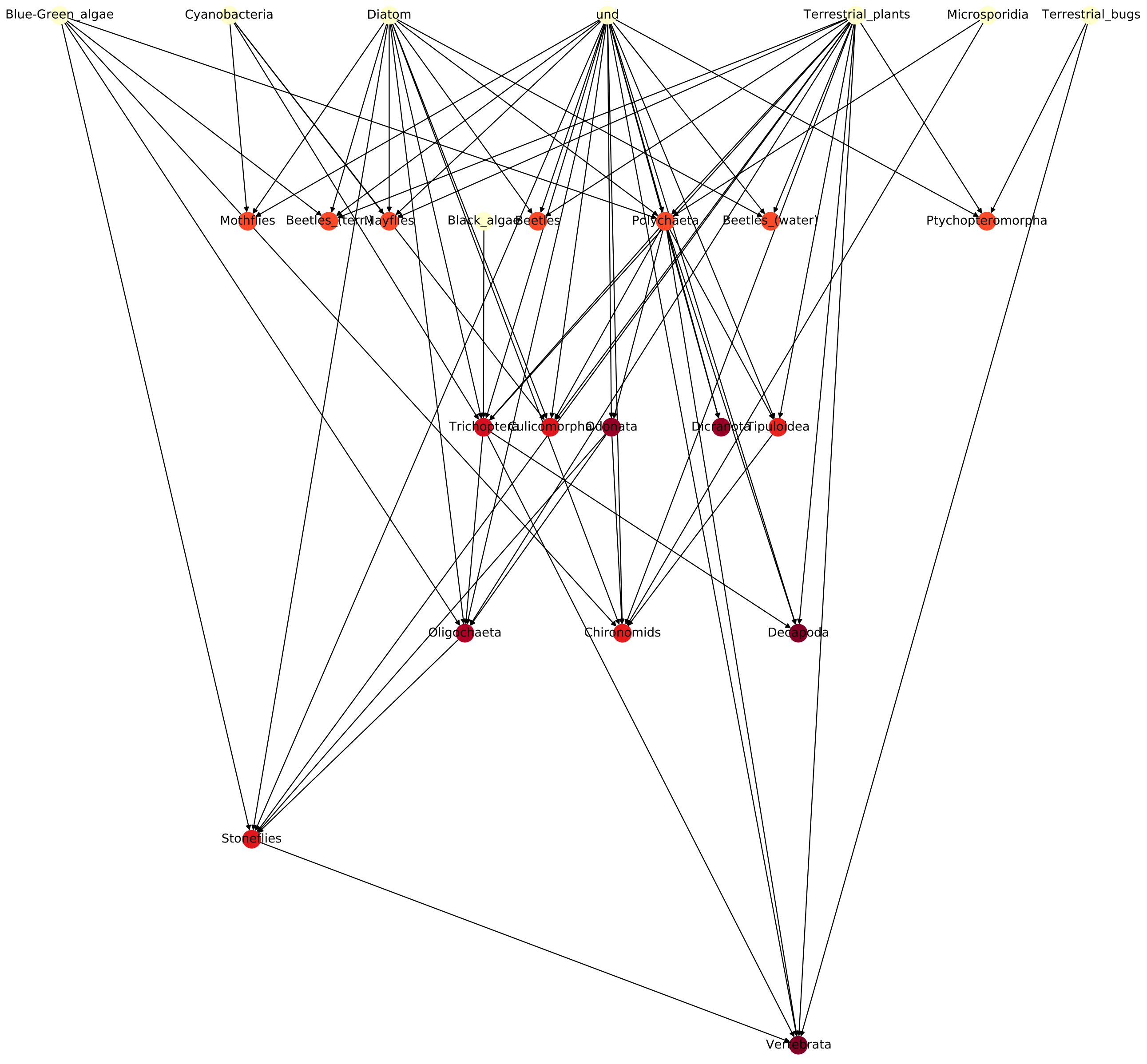

Figure S6: North Carolina Grouped food web: Tree hierarchy

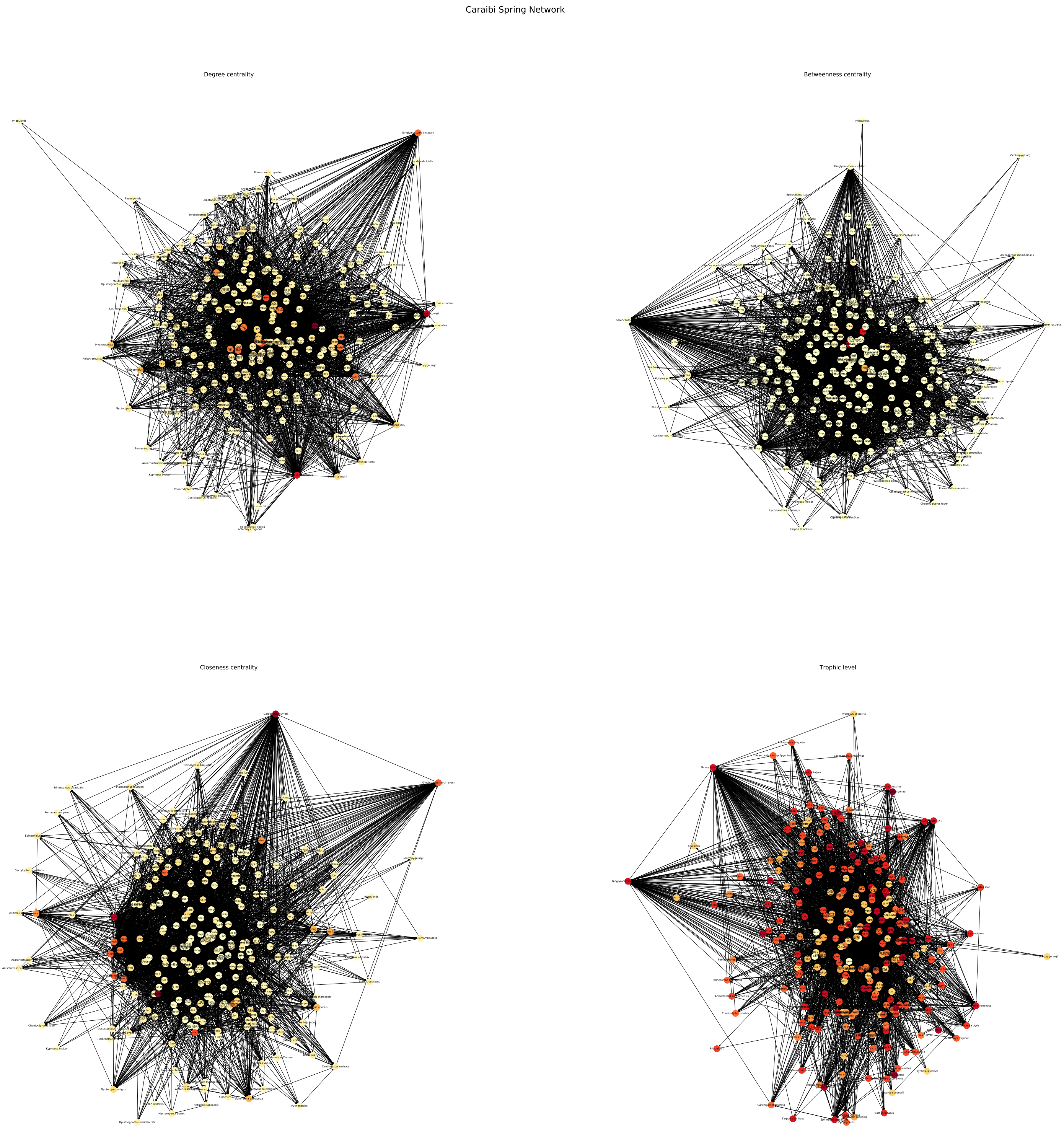

Figure S7: Caribbean RAW food web: Spring layout

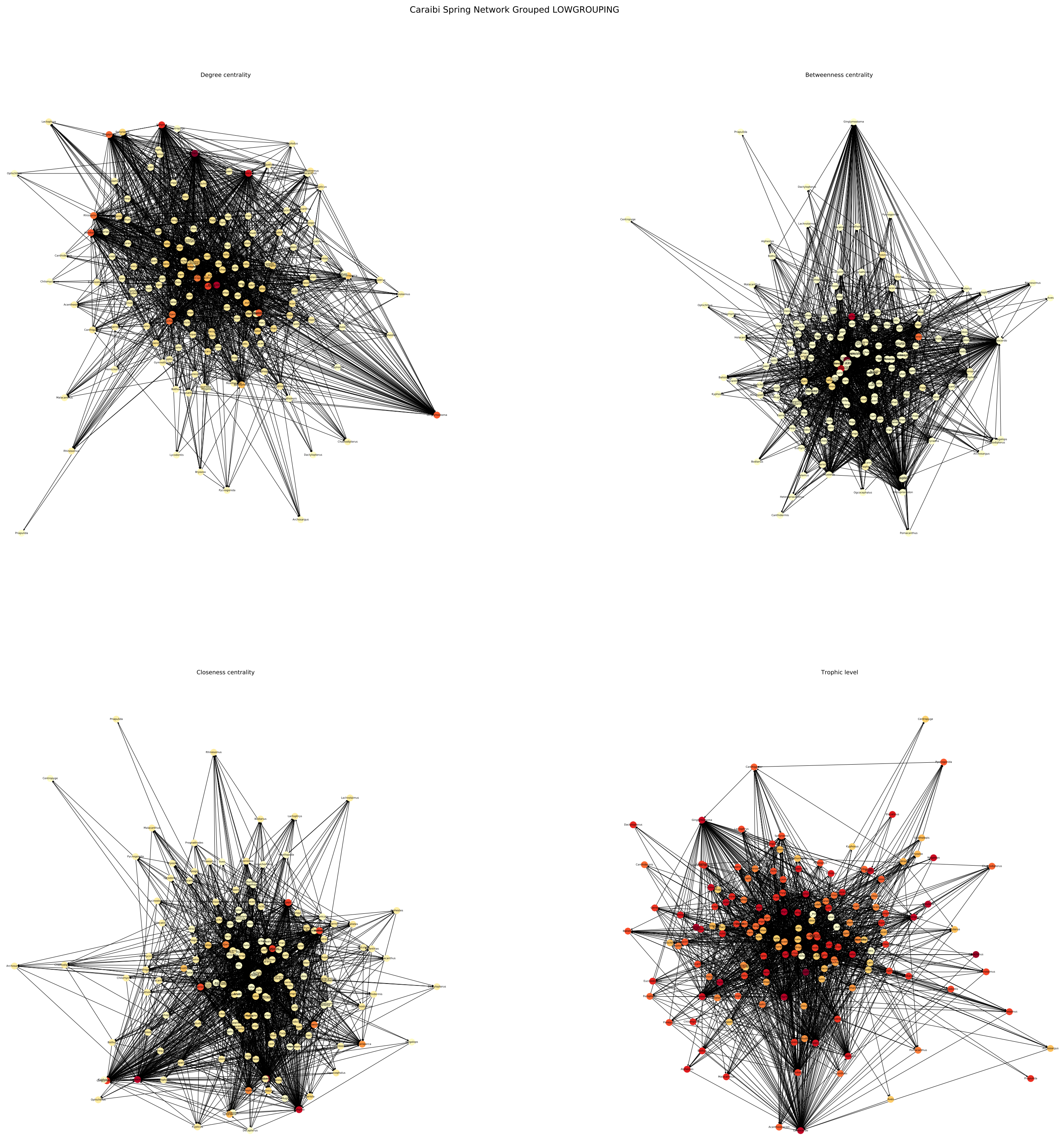

Figure S8: Caribbean LOW food web: Spring layout

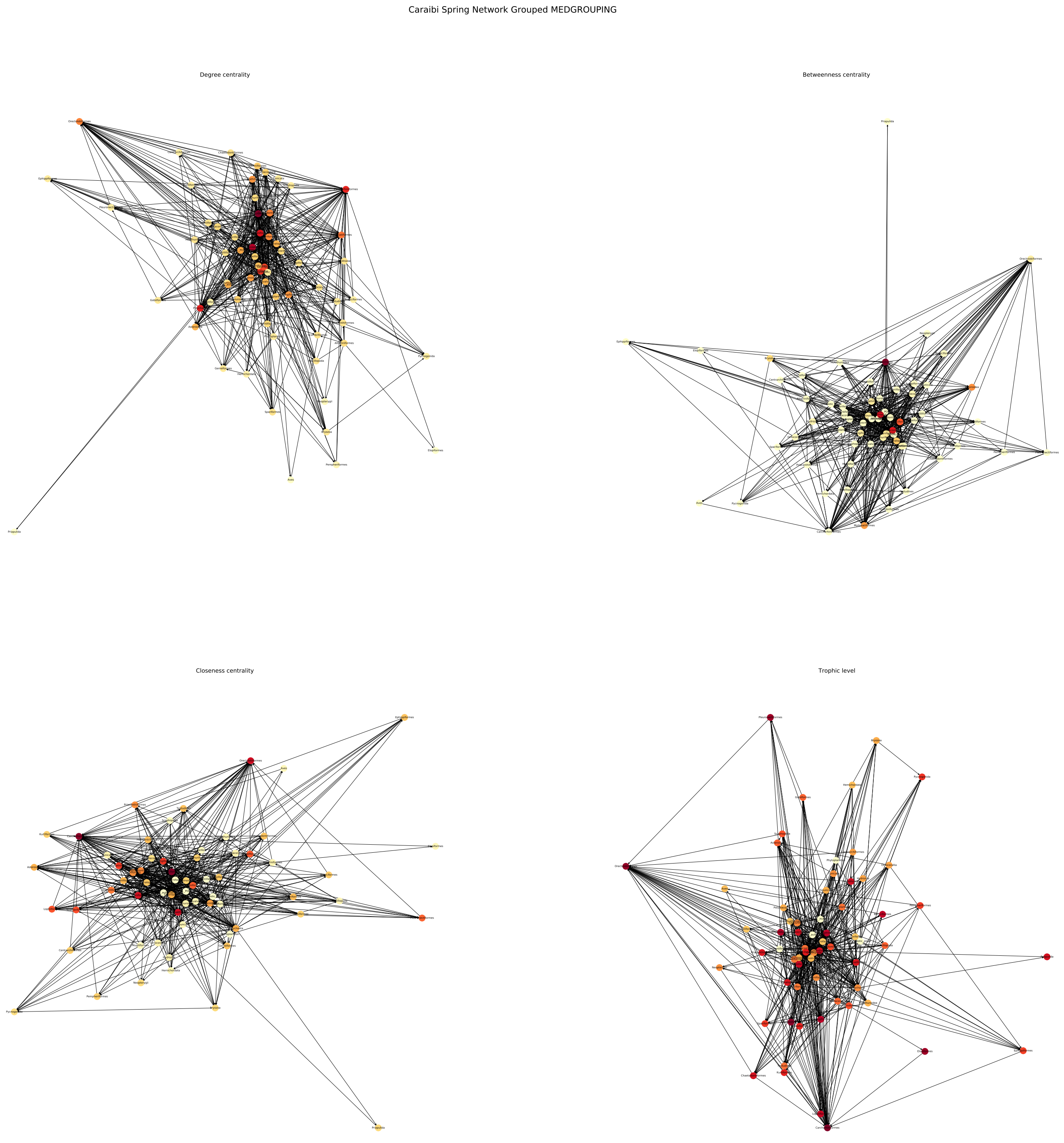

Figure S9: Caribbean MED food web: Spring layout

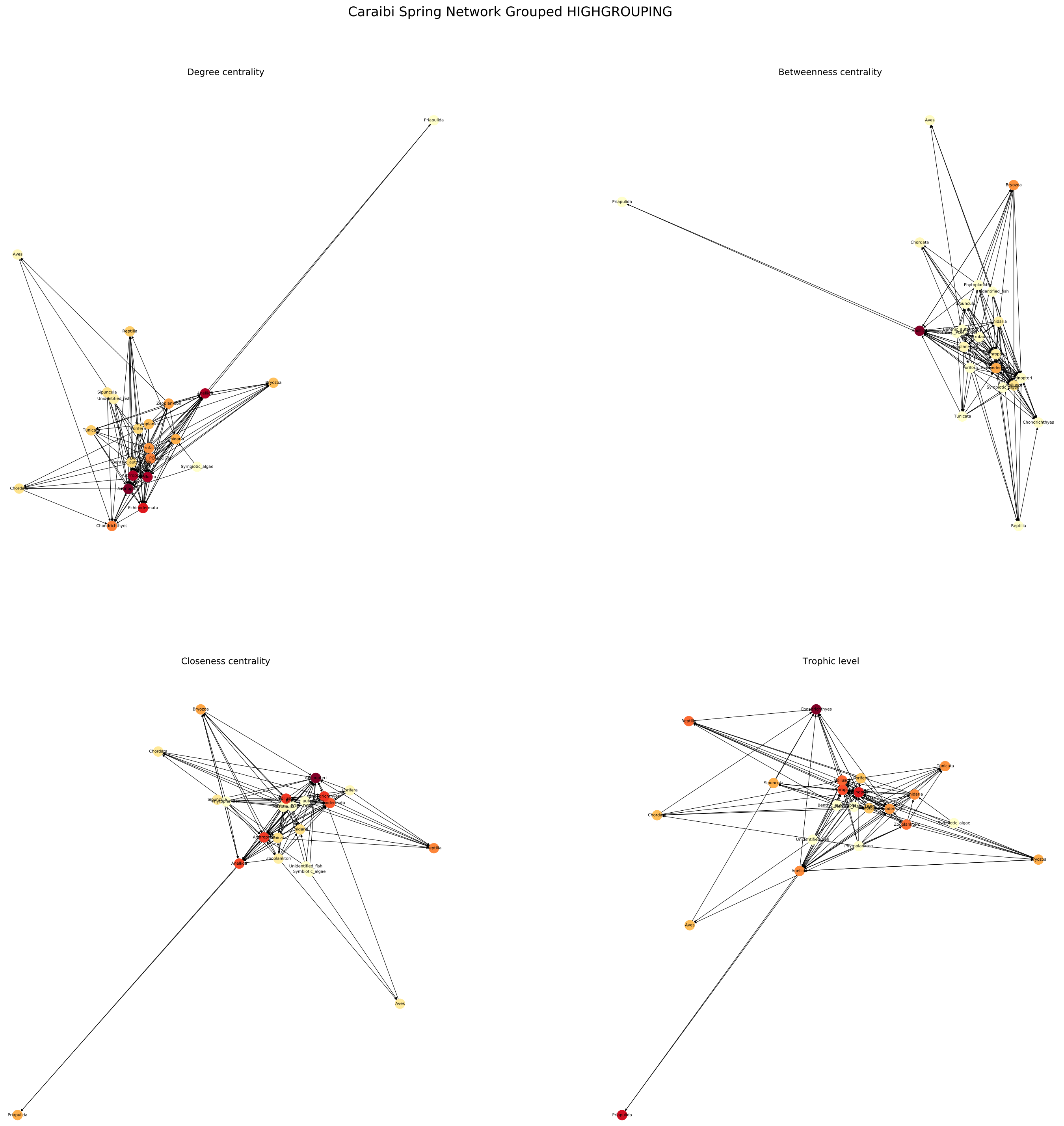

Figure S10: Caribbean HIGH food web: Spring layout

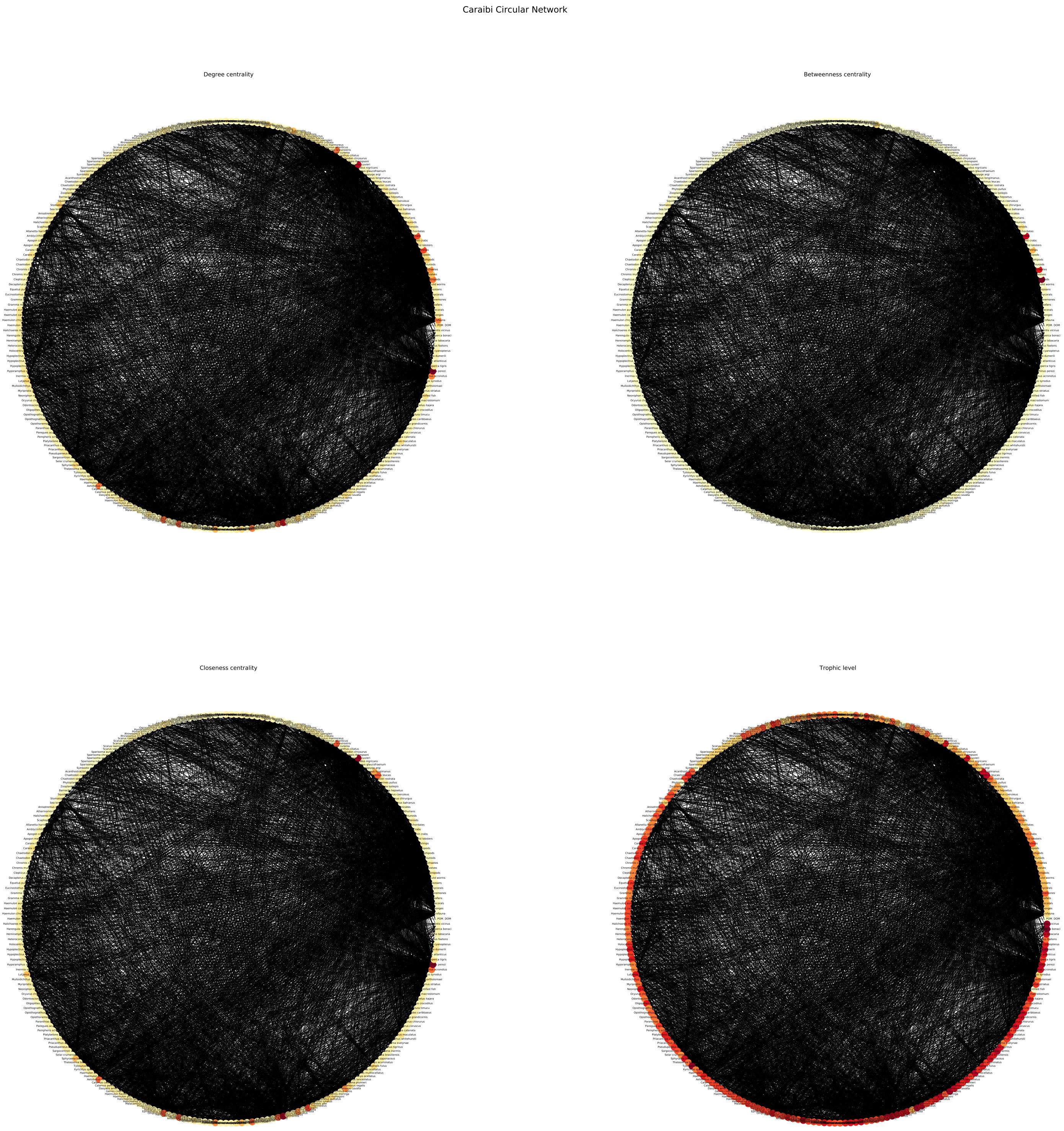

Figure S11: Caribbean RAW food web: Circular layout

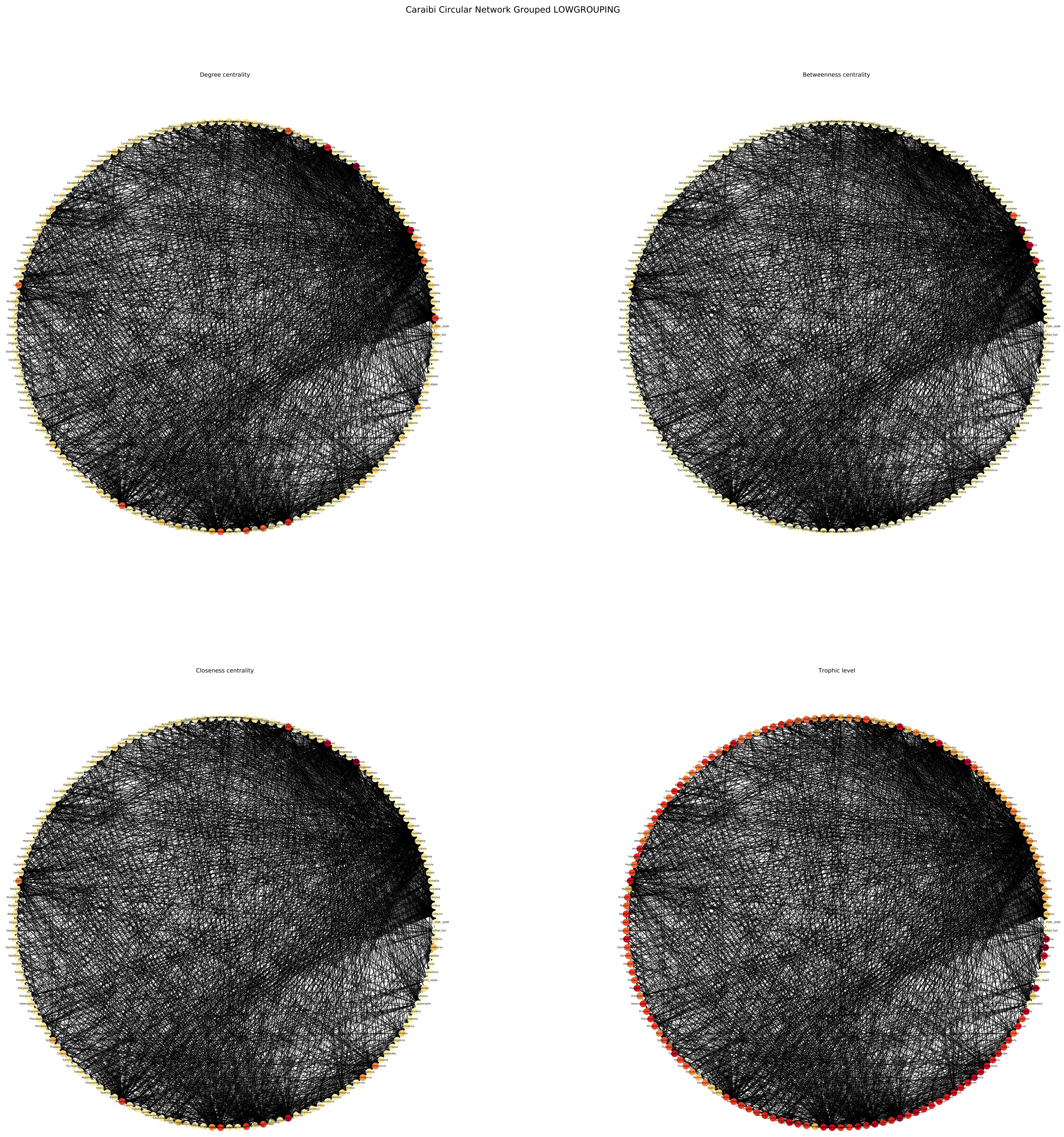

Figure S12: Caribbean LOW food web: Circular layout

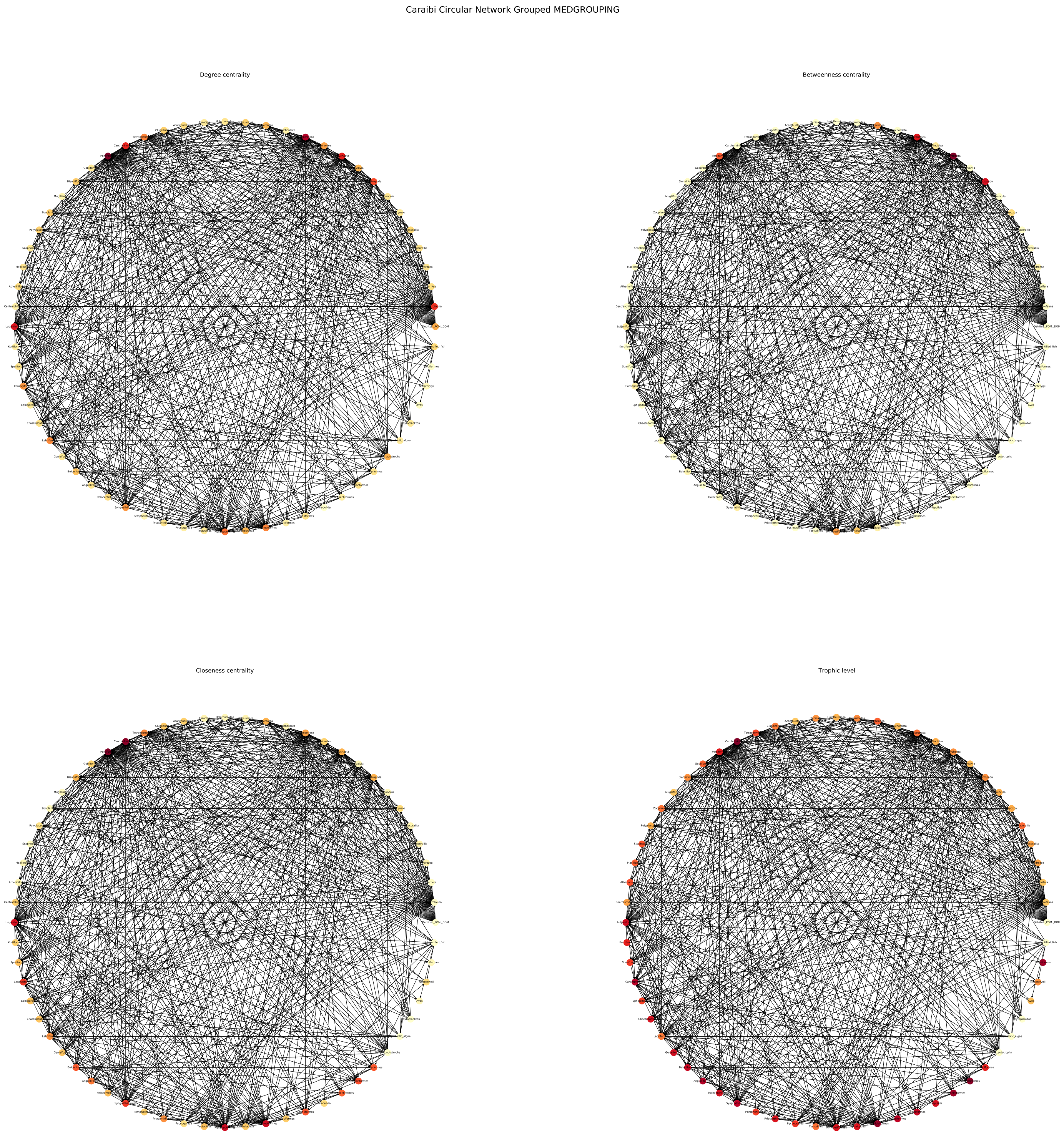

Figure S13: Caribbean MED food web: Circular layout

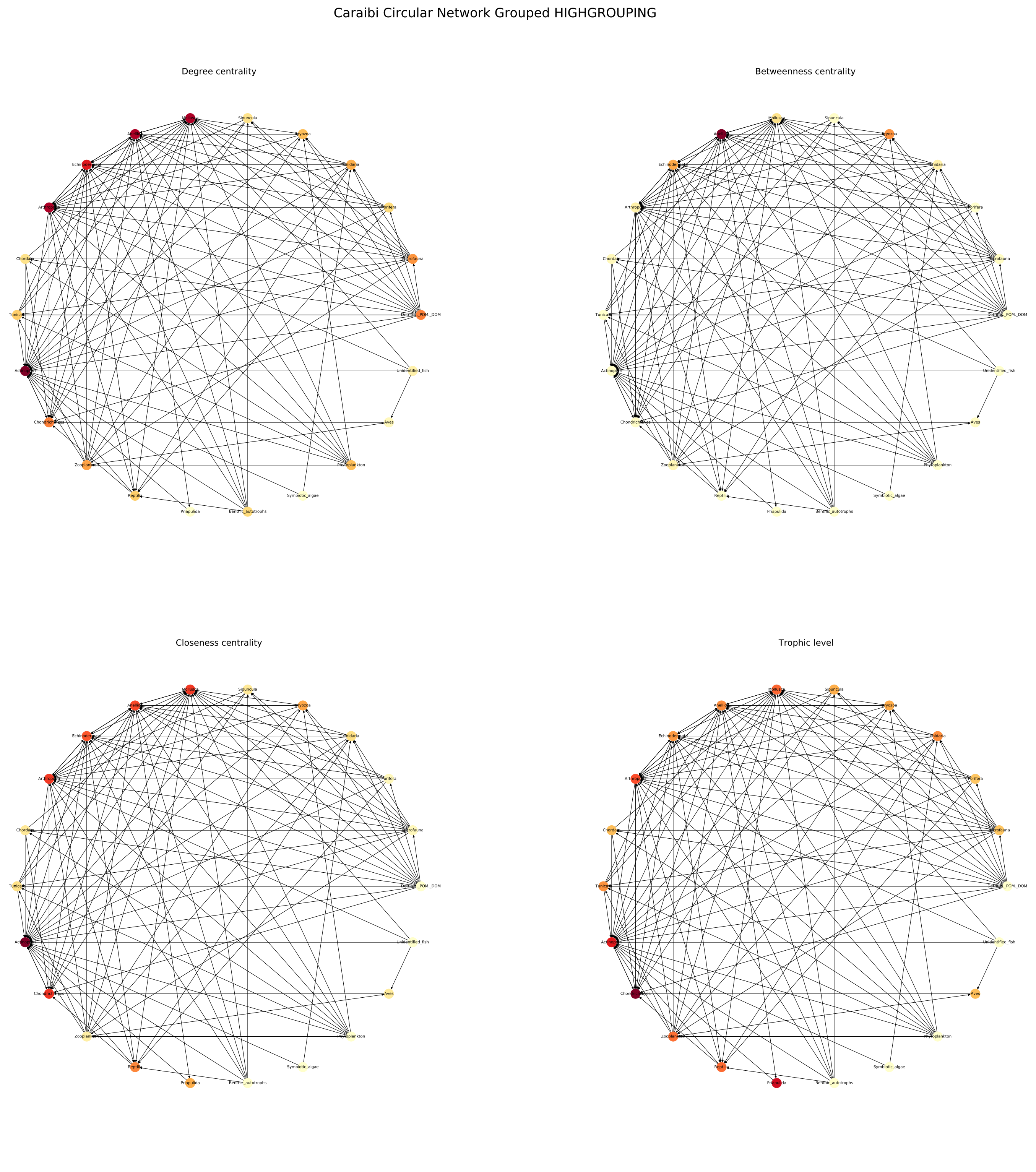

Figure S14: Caribbean HIGH food web: Circular layout

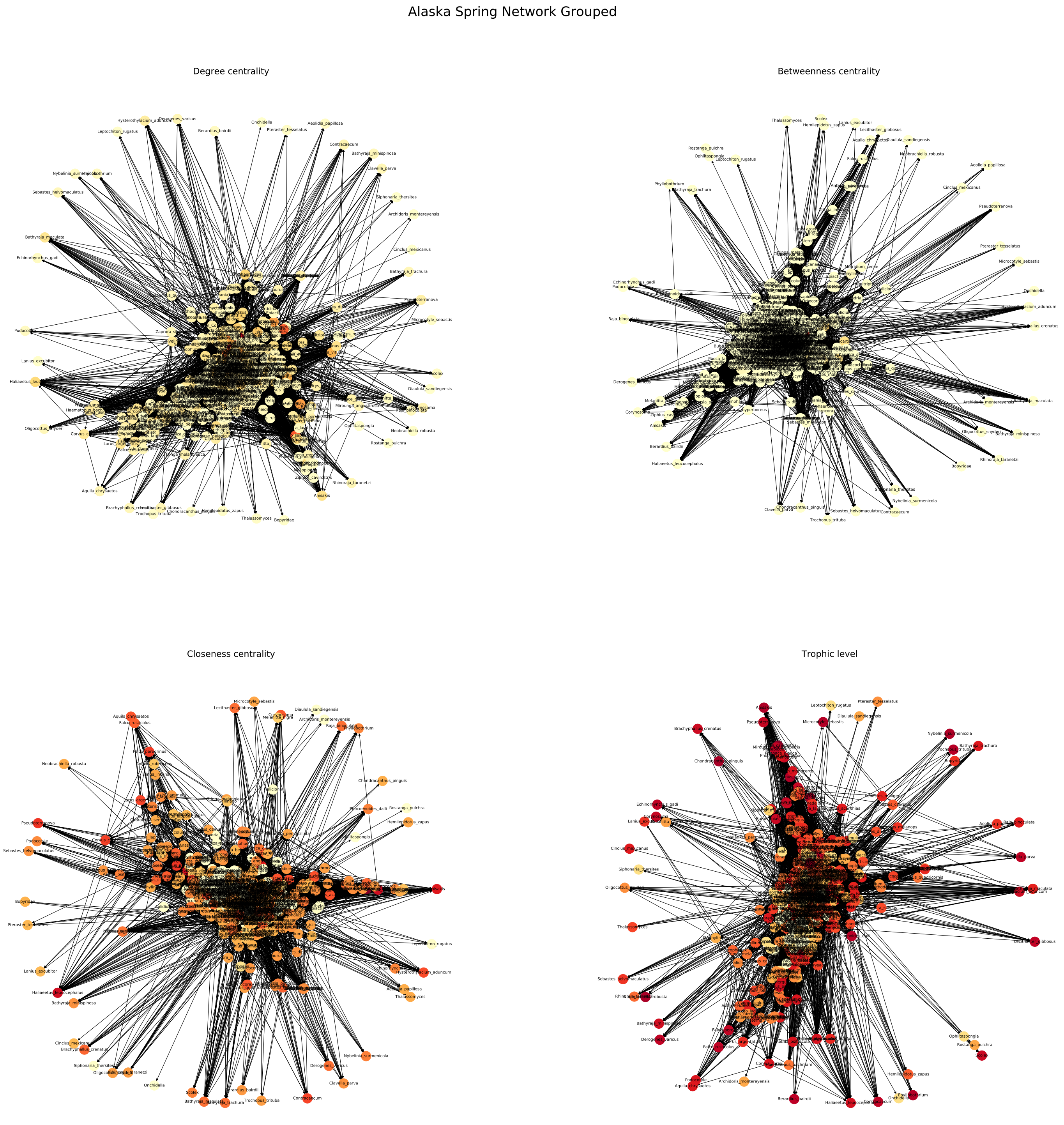

Figure S15: Alaska RAW food web: Spring layout

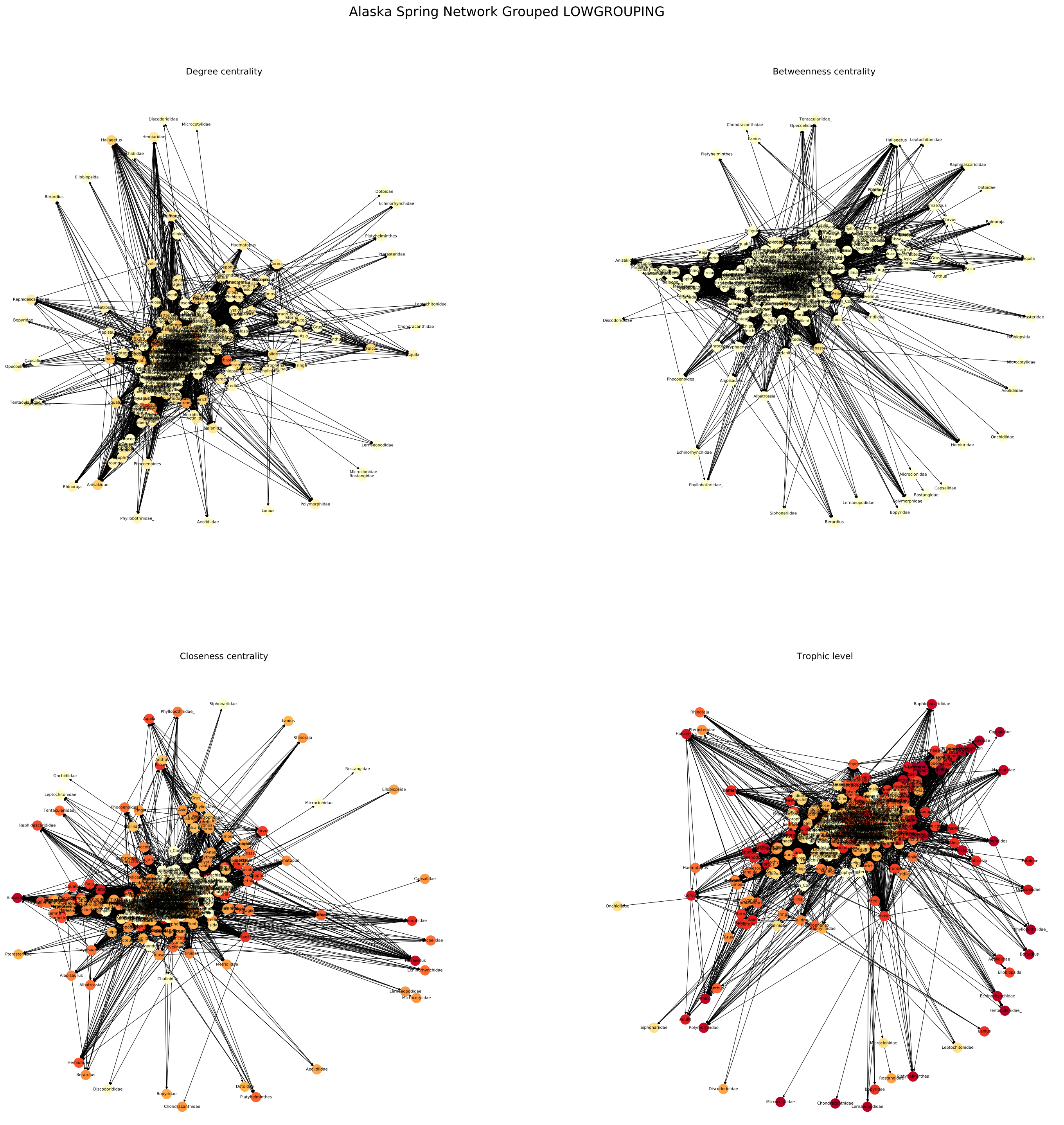

Figure S16: Alaska LOW food web: Spring layout

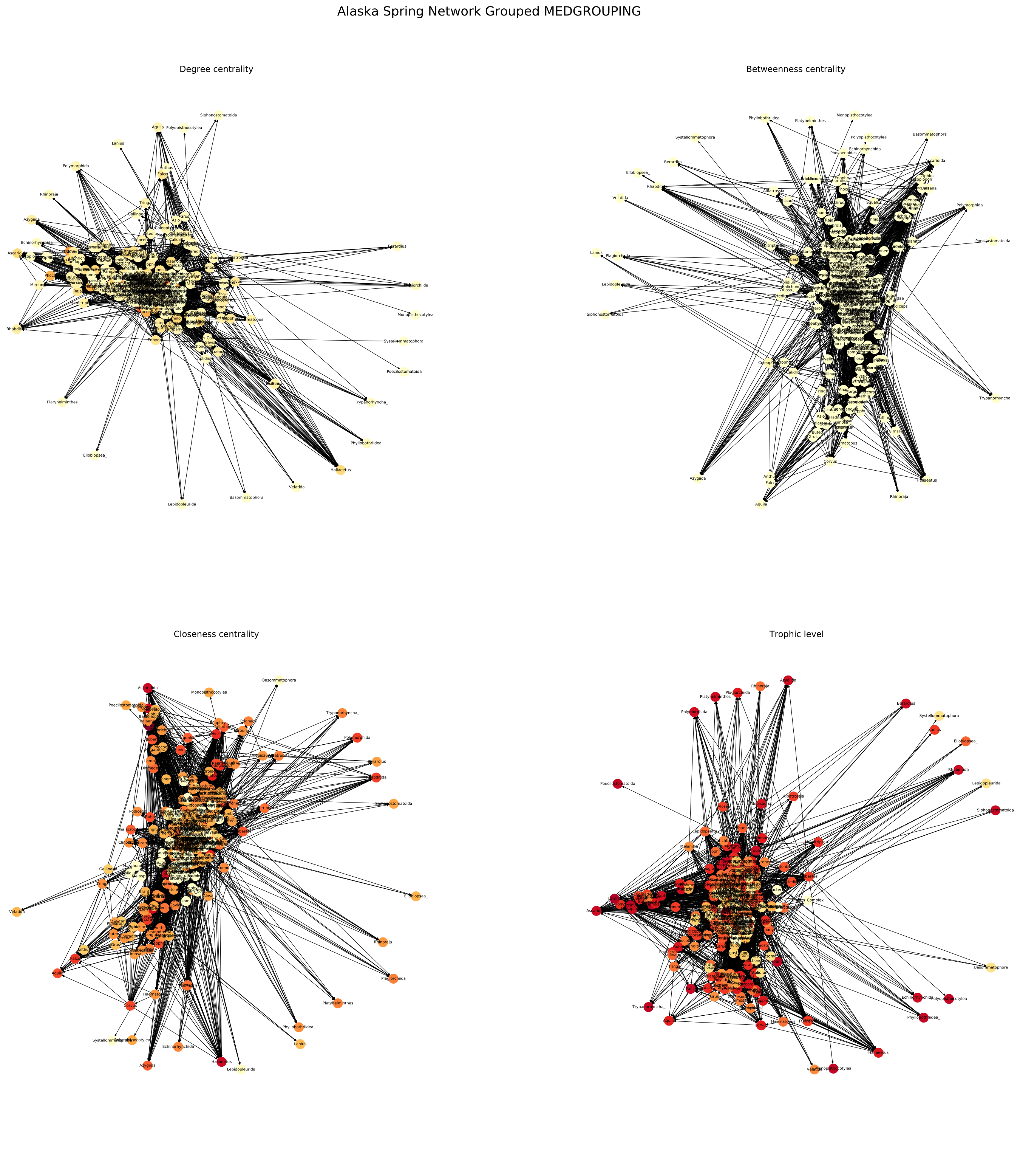

Figure S17: Alaska MED food web: Spring layout

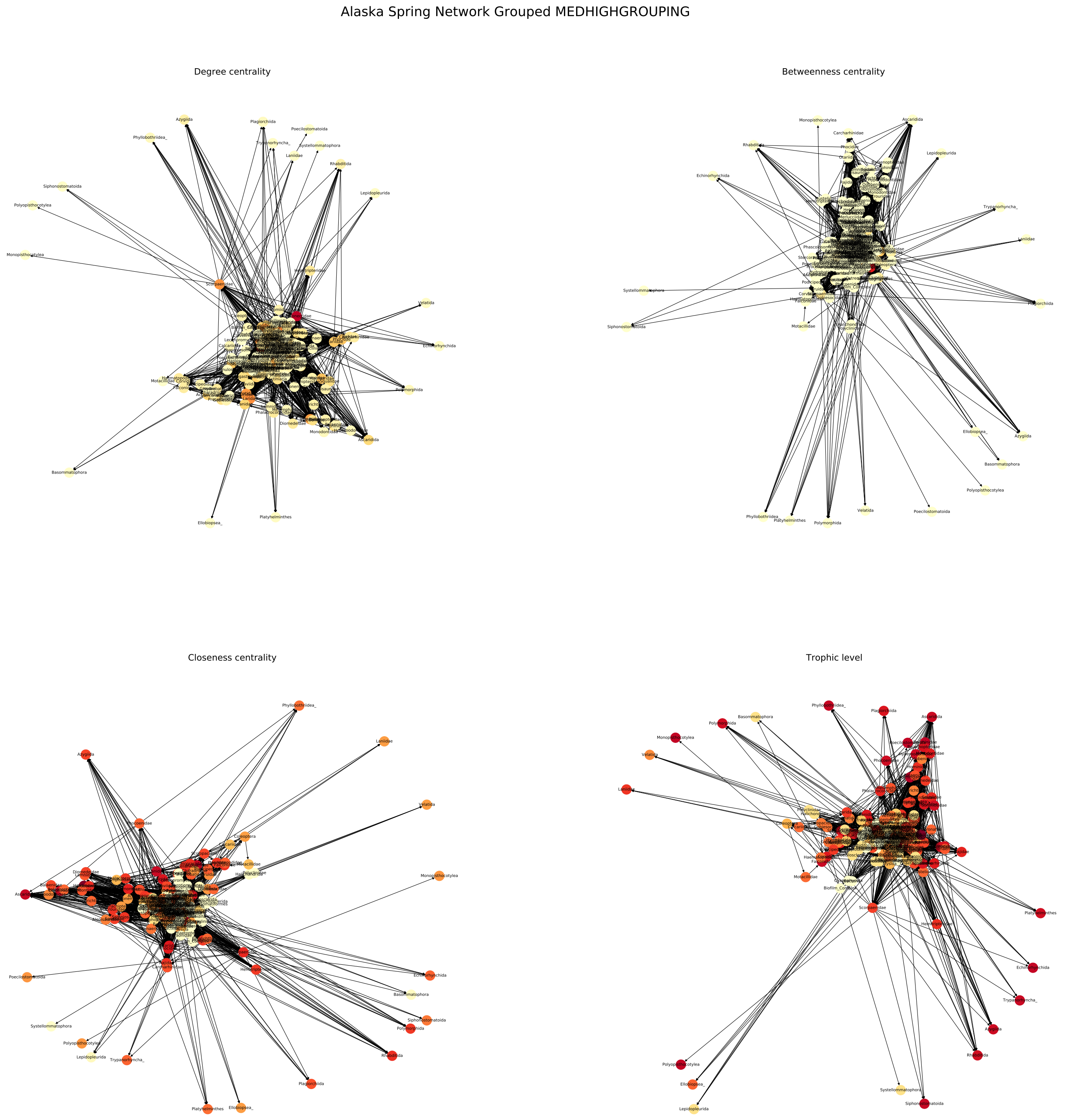

Figure S18: Alaska MEDHIGH food web: Spring layout

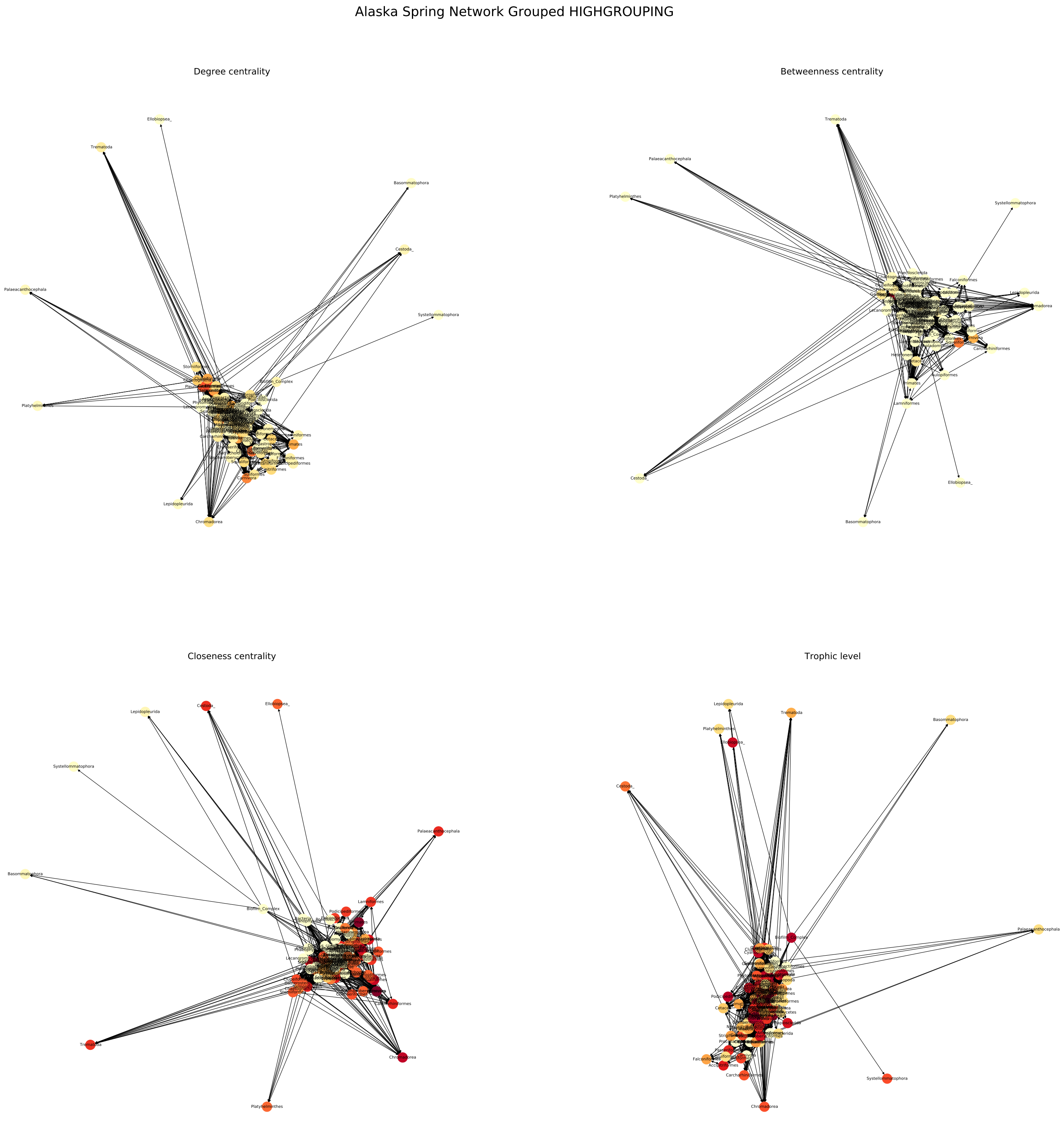

Figure S19: Alaska HIGH food web: Spring layout

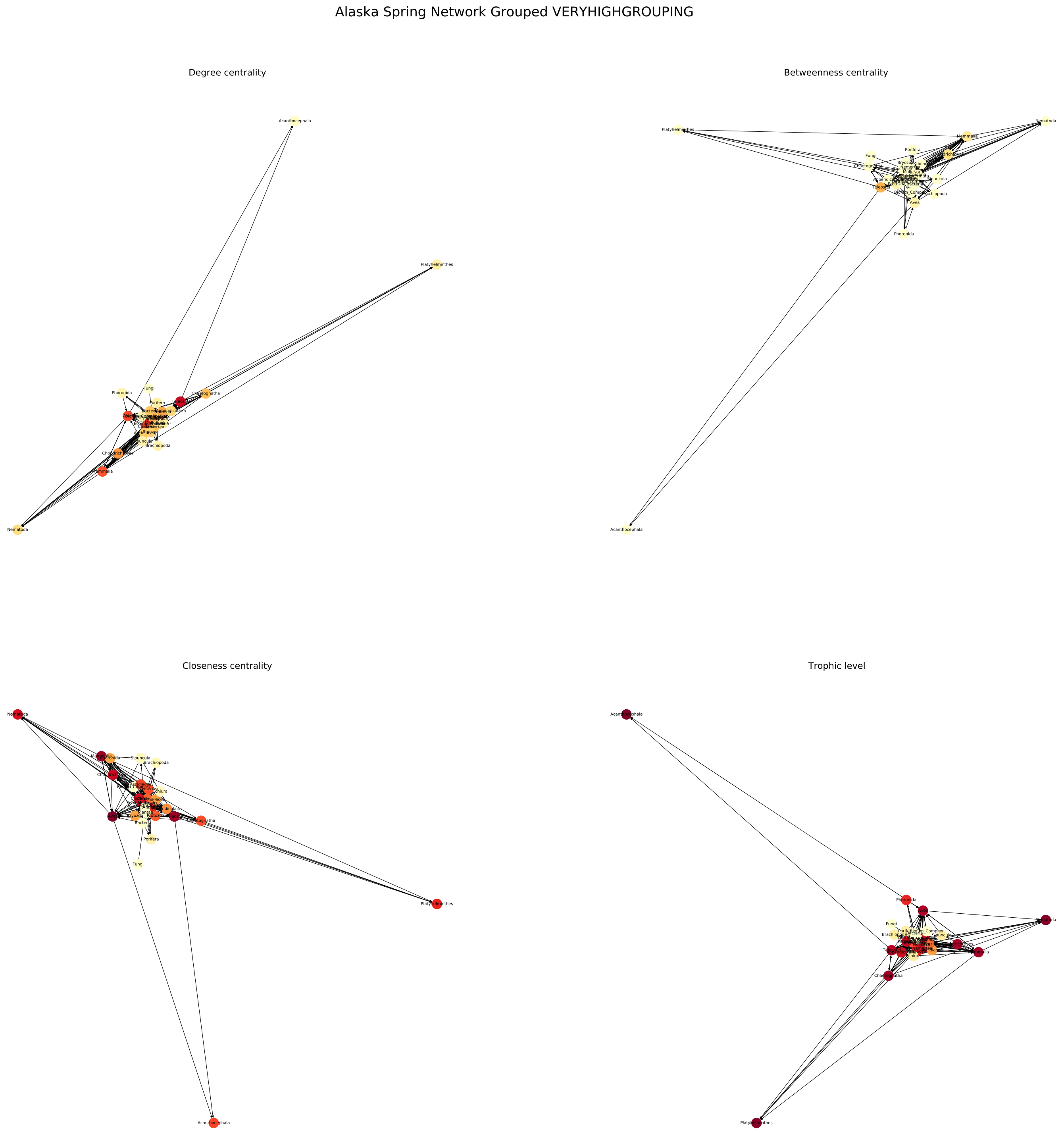

Figure S20: Alaska TOP food web: Spring layout

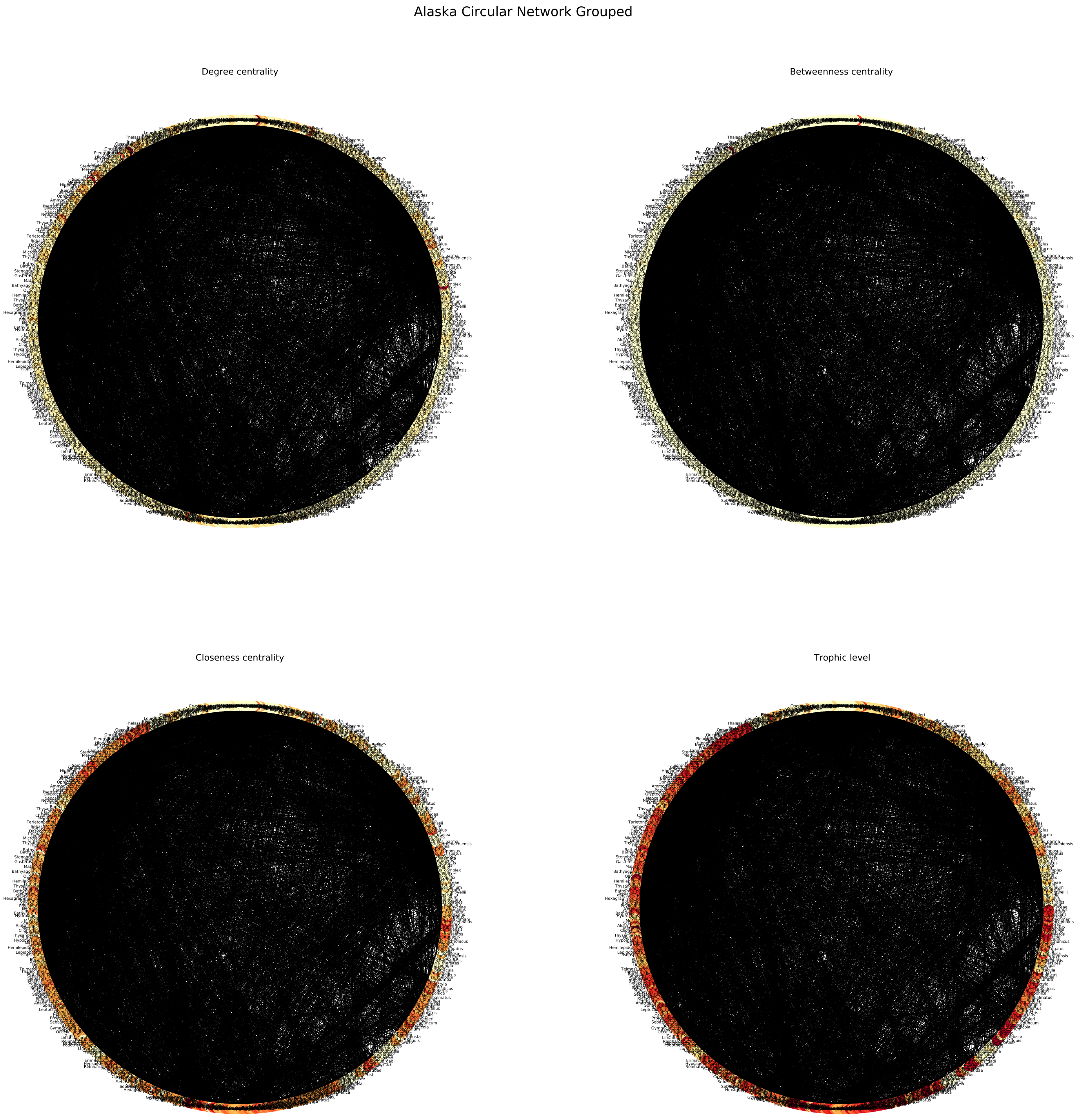

Figure S21: Alaska RAW food web: Circular layout

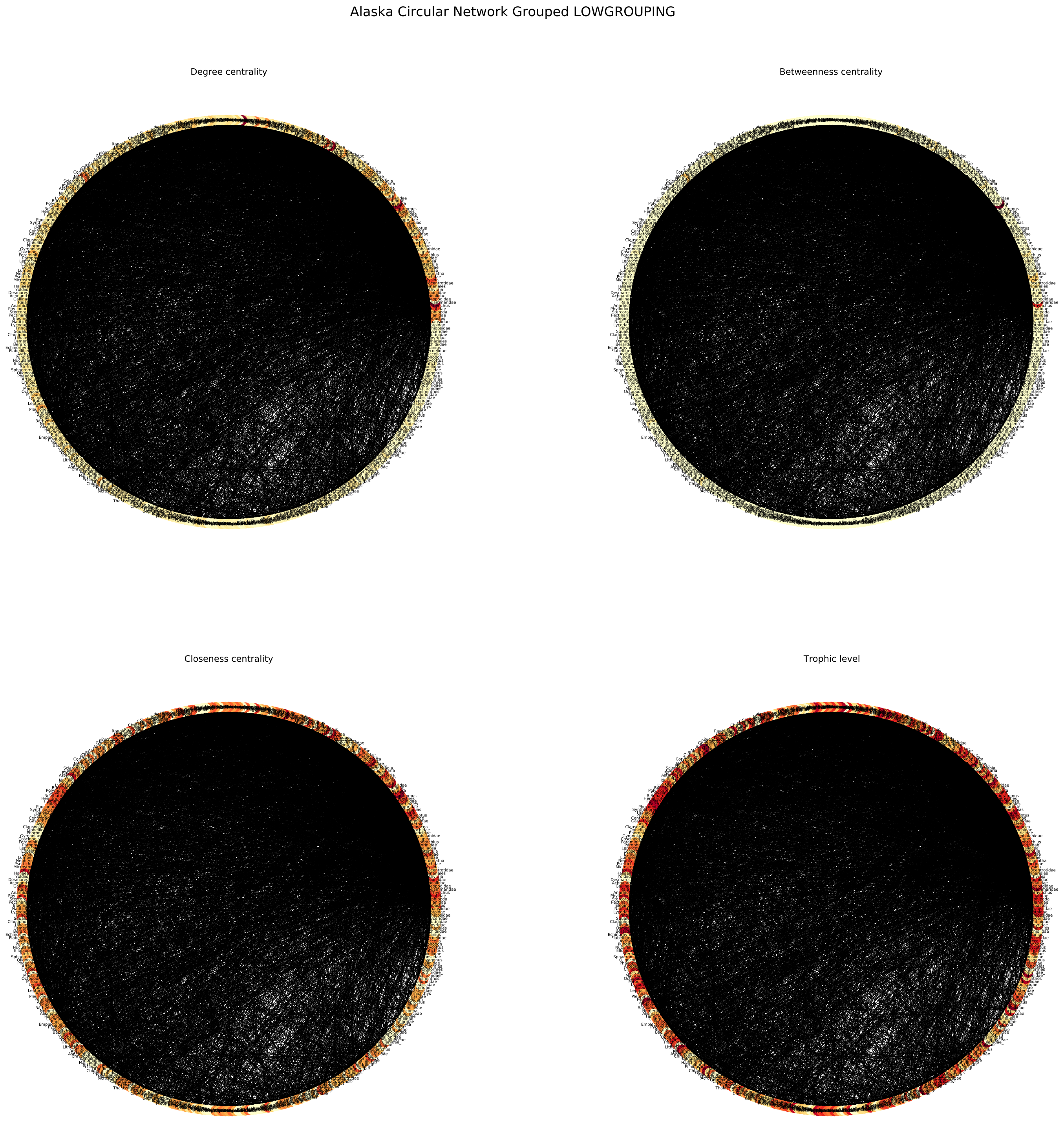

Figure S22: Alaska LOW food web: Circular layout

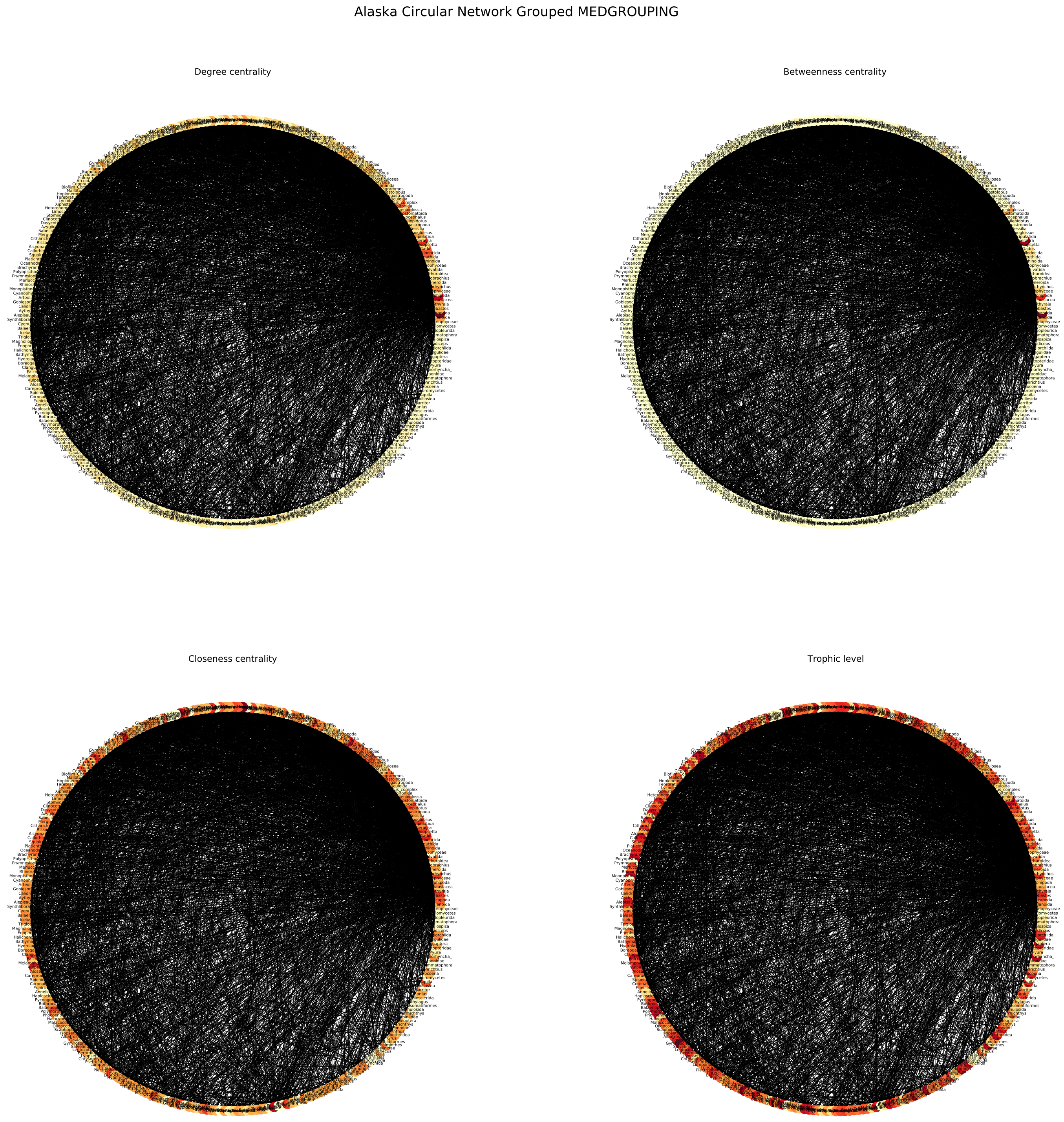

Figure S23: Alaska MED food web: Circular layout

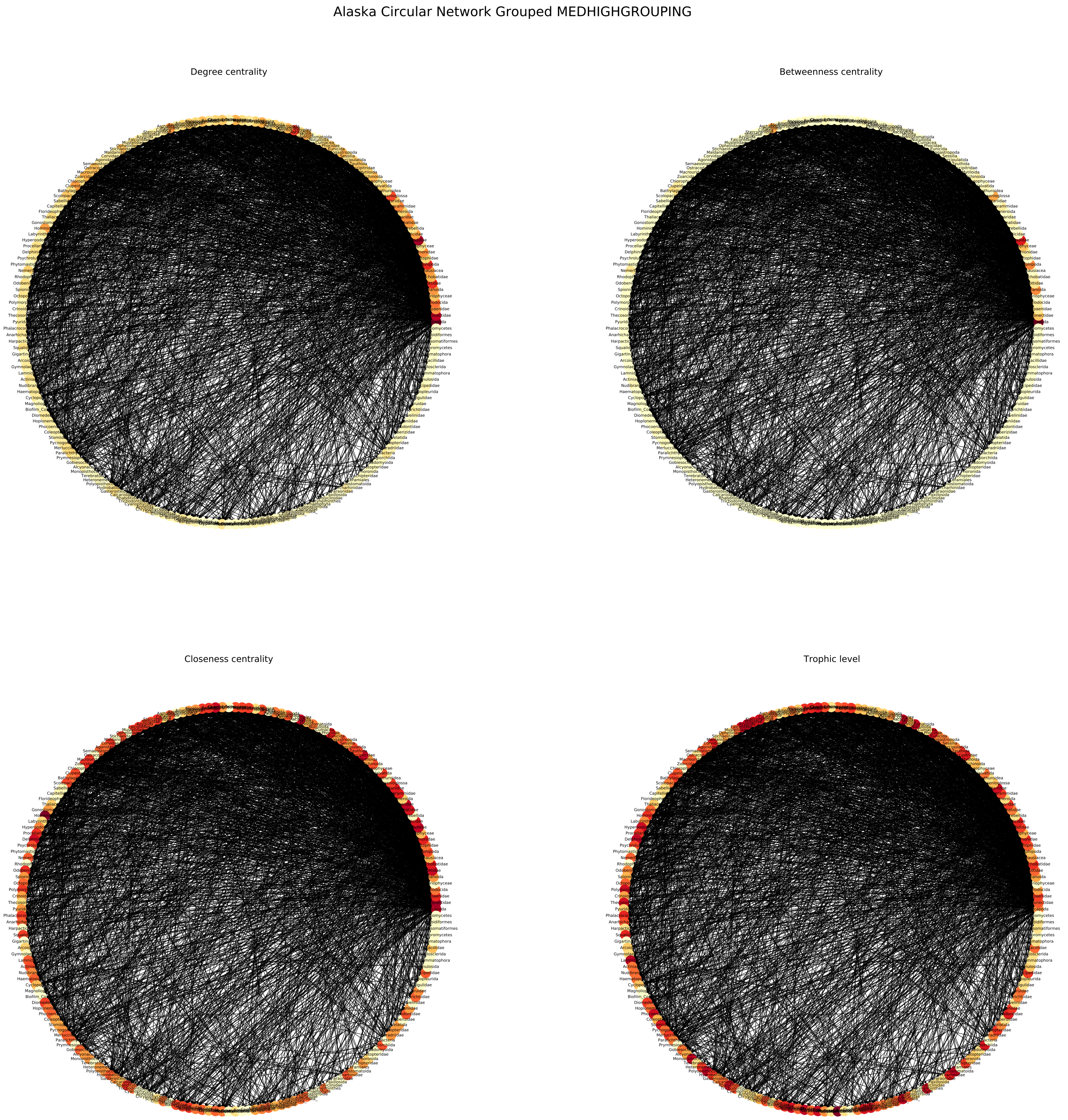

Figure S24: Alaska MEDHIGH food web: Circular layout

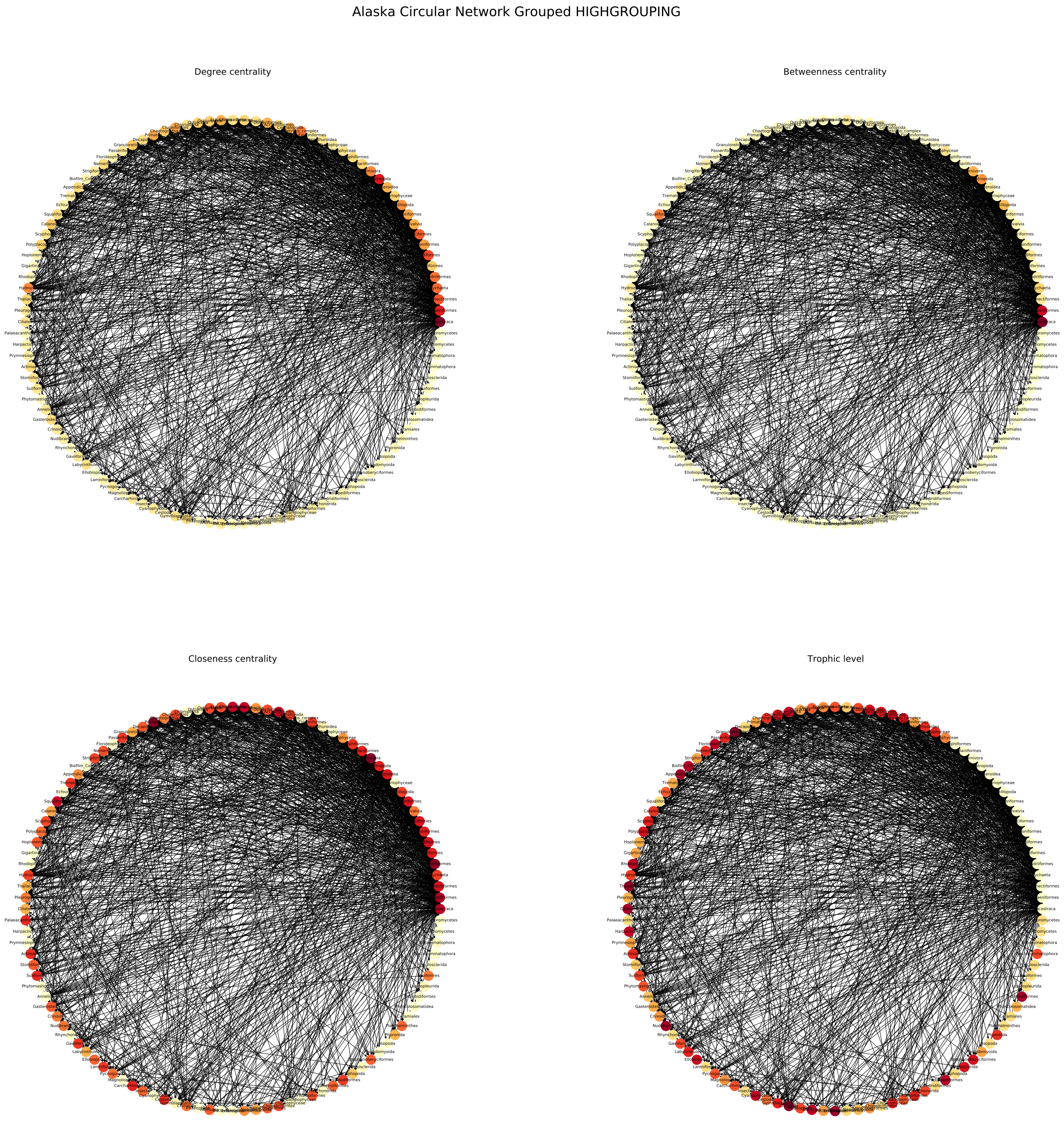

Figure S25: Alaska HIGH food web: Circular layout

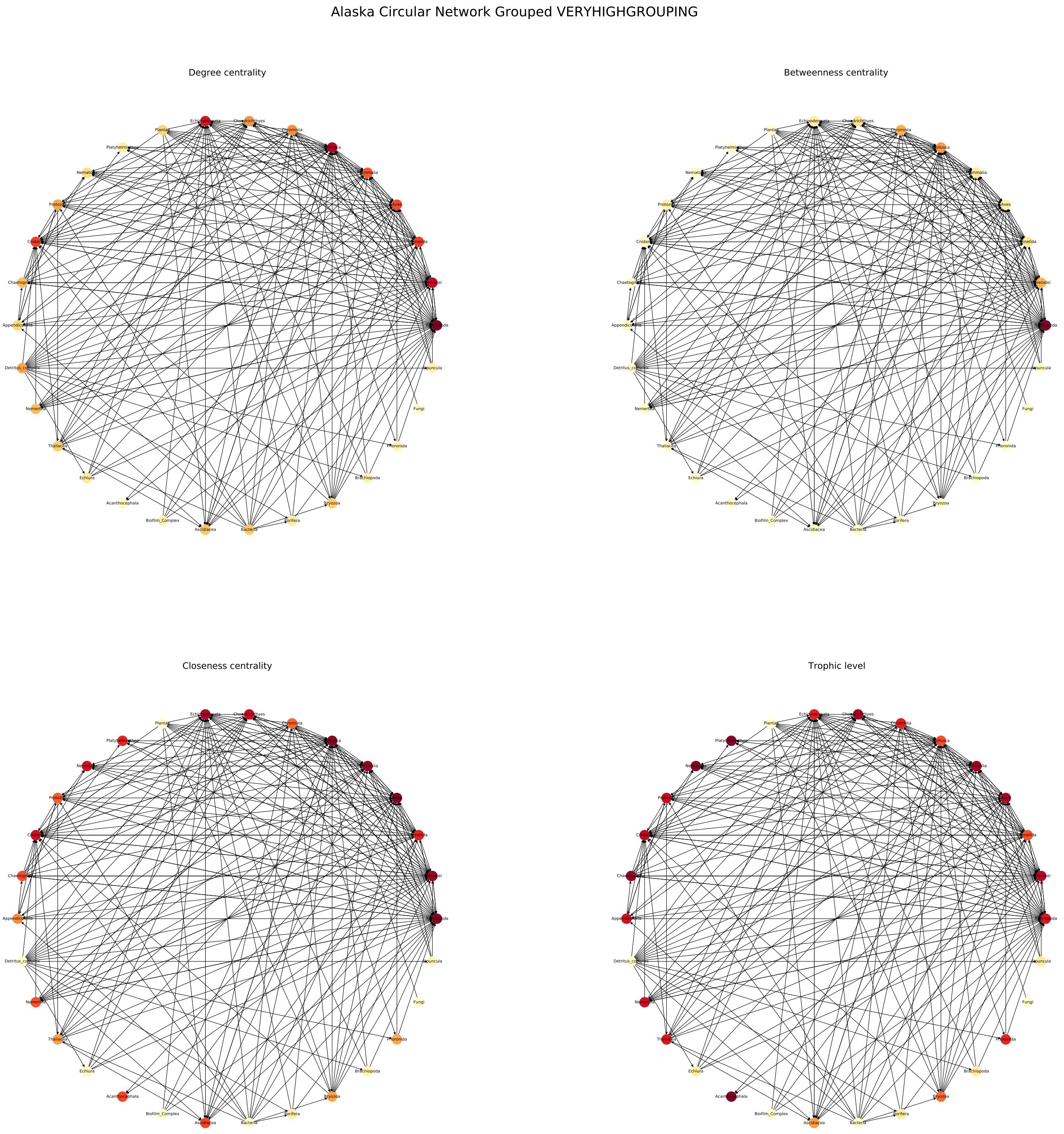

Figure S26: Alaska TOP food web: Circular layout
