# Supplementary material for: A network simplification approach to ease topological studies about the food-web architecture
Source: Sci Rep. 2022 Aug 17;12:13948. doi: 10.1038/s41598-022-17508-1 (PMC9385703; doi:10.1038/s41598-022-17508-1)
Supplement: Supplementary file 2 — Supplementary Information 2. [file 41598_2022_17508_MOESM2_ESM.zip › Node_grouping_by_degree.html]

Figure S38: North Carolina Sankey graph for node grouping by degree

Figure S38: North Carolina Sankey graph for node grouping by degree. The first column shows the node in the original network, the second one represents the grouping action and the degree value is the sum of the degrees, the third one shows the mean value of the degree
