# Supplementary material for: A network simplification approach to ease topological studies about the food-web architecture
Source: Sci Rep. 2022 Aug 17;12:13948. doi: 10.1038/s41598-022-17508-1 (PMC9385703; doi:10.1038/s41598-022-17508-1)
Supplement: Supplementary file 2 — Supplementary Information 2. [file 41598_2022_17508_MOESM2_ESM.zip › Node_grouping_by_Girvan-Newman.html]

Figure S39: North Carolina Sankey graph for node grouping by Girvan-Newman algorithm

Figure S39: North Carolina Sankey graph for node grouping by Girvan-Newman algorithm. The first and third columns show respectively the un-aggregated and aggregated nodes with a number that shows how many entities where grouped (the first column in un-aggregated and so the number equal 1). The second and fourth columns shows the result fo the Girvan-Newman community detection algorithm on the original amd simplified network in clusters (the number is again the sum of entities in a cluster).
