# Supplementary material for: A network simplification approach to ease topological studies about the food-web architecture
Source: Sci Rep. 2022 Aug 17;12:13948. doi: 10.1038/s41598-022-17508-1 (PMC9385703; doi:10.1038/s41598-022-17508-1)
Supplement: Supplementary file 2 — Supplementary Information 2. [file 41598_2022_17508_MOESM2_ESM.zip › Node_grouping_by_Infomap_modularity.html]

Figure S40: North Carolina Sankey graph for node grouping by Infomap modularity

Figure S40: North Carolina Sankey graph for node grouping by Infomap modularity. The first column shows the original node list and the values of the infomap algorithm towards clusters indicated in the second column (with a number representing the number of nodes in a particular cluster). The third column shows the aggregated nodes with a number that sum the nodes that were grouped, that go inside clusters of the fourth column (the number here represent the values of the algorithm). In this way the middle part of the plot shows the differences in clusterisation between the original ans simplified network, and the edges represent the values of the Infomap algorithm between first and second column.
