# Supplementary material for: A network simplification approach to ease topological studies about the food-web architecture
Source: Sci Rep. 2022 Aug 17;12:13948. doi: 10.1038/s41598-022-17508-1 (PMC9385703; doi:10.1038/s41598-022-17508-1)
Supplement: Supplementary file 2 — Supplementary Information 2. [file 41598_2022_17508_MOESM2_ESM.zip › Node_grouping_by_trophic_level.html]

Figure S42: North Carolina Sankey graph for node grouping by trophic level

Figure S42: North Carolina Sankey graph for node grouping by trophic level. The first column shows the original node list and the values of trophic index towards clusters indicated in the second column (the cluster is a group of nodes with the same trophic index indicated by the number after the letter). The third and fourth columns show the same information for the simplified network.
