# Supplementary material for: A network simplification approach to ease topological studies about the food-web architecture
Source: Sci Rep. 2022 Aug 17;12:13948. doi: 10.1038/s41598-022-17508-1 (PMC9385703; doi:10.1038/s41598-022-17508-1)
Supplement: Supplementary file 3 — Supplementary Information 3. [file 41598_2022_17508_MOESM3_ESM.pdf]

	North Carolina		Caraibi				Alaska					
	raw	med	raw	low	med	high	Raw	low	med	med-hi	high	top
Average clustering	0,054	0,008	0,164	0,224	0,306	0,395	0,128	0,157	0,214	0,270	0,362	0,509
Network hierarchy	1,000	1,000	0,985	0,978	0,872	0,803	0,625	0,607	0,600	0,585	0,335	0,338

Table S1: Summary information on clustering and hierarchy for the three networks analysed per level of simplification

---

	Graph cumulated degree	Graph mean degree	Grouped Graph degree
Beetles	4	4	3
Beetles_(terr.)	6	6	4
Beetles_(water)	15	15	3
Black_algae	1	1	1
Blue-Green_algae	5	2,5	5
Chironomids	16	3,2	7
Culicomorpha	5	5	5
Cyanobacteria	4	2	4
Decapoda	4	4	4
Diatom	59	2,1	11
Dicranota	2	2	2
Mayflies	27	9	4
Microsporidia	2	2	2
Mothflies	4	4	3
Odonata	8	4	5
Oligochaeta	11	5,5	7
Polychaeta	18	18	12
Ptychopteromorpha	3	3	3
Stoneflies	16	5,3	7
Terrestrial_bugs	2	2	2
Terrestrial_plants	22	22	14
Tipuloidea	8	2, 7	4
Trichoptera	15	3	9
Vertebrata	6	6	6
und	33	16,5	17

Table S2: Node degree for the North Carolina network before and after the taxonomical grouping. The cumulated degree refers to the sum of the degrees of each node in the raw network per taxonomical group; the mean degree is the mean of the degree of the raw network based on the number of entities of that group (e.g.: Diatom: cumulated degree divided by the number of diatoms); the grouped graph degree in the recomputed degree in the simplified network in which each group is treated as a node.

Reference	Index or measure
44	DC, BC, CC, EC, Clustering coefficient
45	DC, BC
46	CC, KC
47	DC, BC, CC, EC
48	DC, KC

Table S3: Summary of recent ecological with the list of computed indices and measurements.
In the first column we reported already cited references; in the second column we listed the nodel level indeces and general measurement used in these articles and chosen in our approach.
