# Supplementary figures and images for: A network simplification approach to ease topological studies about the food-web architecture
Source: Sci Rep. 2022 Aug 17;12:13948. doi: 10.1038/s41598-022-17508-1 (PMC9385703; doi:10.1038/s41598-022-17508-1)

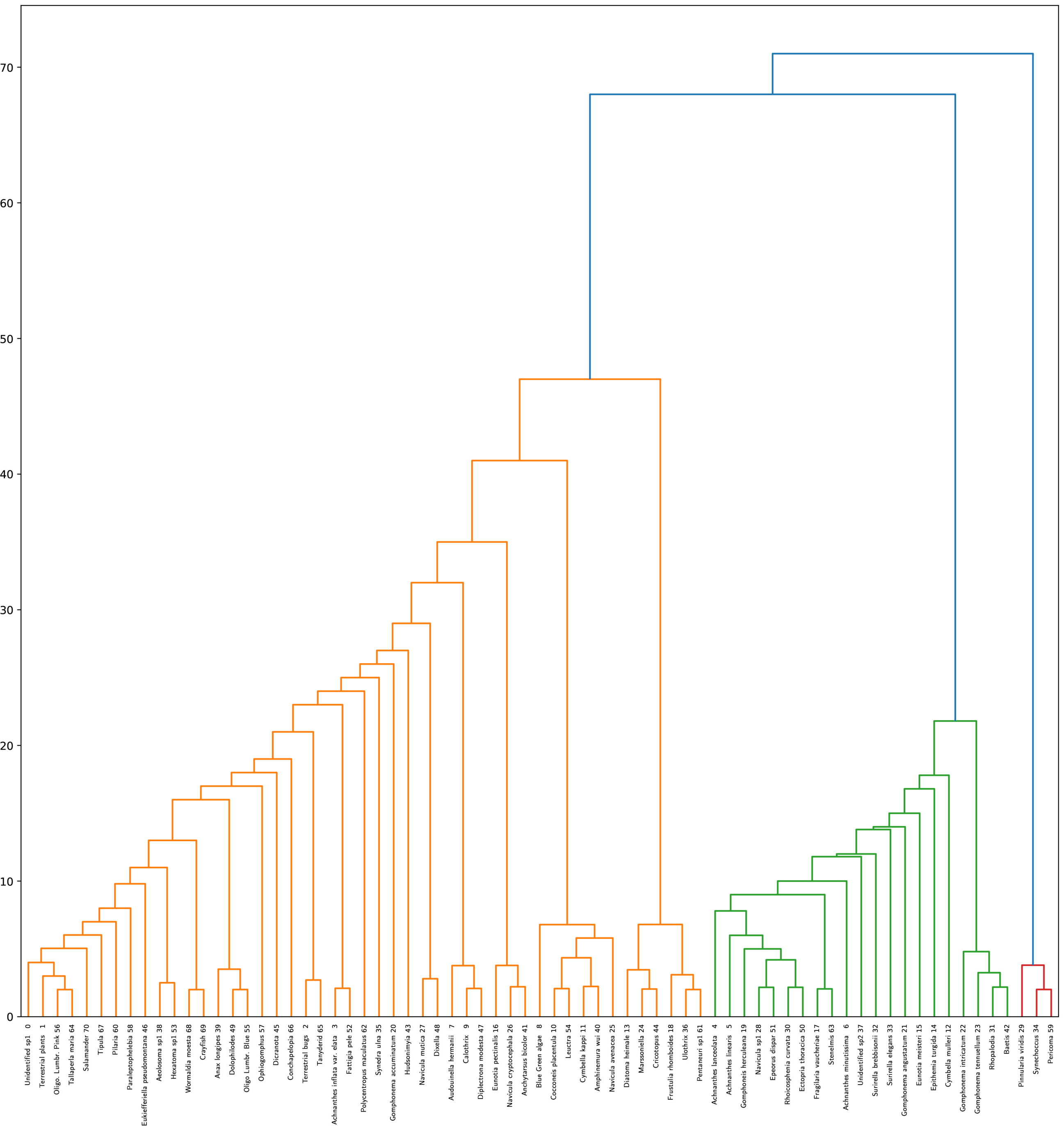

Figure S35: North Carolina raw network dendrogram for the Girvan-Newman community detection

Supplement: Supplementary file 4 — Supplementary Information 4. [file 41598_2022_17508_MOESM4_ESM.zip › NC_dendrogram_graph.pdf]
